# Cultivar Screening and Process Optimization for Quality-Driven Development of Honey-Fermented and Macerated Rose Wines

**DOI:** 10.3390/foods15203597

**Published:** 2026-10-10

**Authors:** Lianqi Cui, Wanwan Liu, Zhe Dong, Jingxuan Wu, Haiwei Zhang, Dongliang Wang

**Affiliations:** 1 College of Horticulture, Anhui Agricultural University, Hefei 230061, China; cuilianqi0620@163.com (L.C.);; 2 School of Food and Nutrition, Anhui Agricultural University, Hefei 230031, China

**Keywords:** edible rose, rose honey-fermented wine, macerated rose wine, SPME-GC×GC-TOF-MS, volatile compounds

## Abstract

Edible roses are rich in sugars, polyphenols, and pigments, but systematic comparisons of cultivar suitability for winemaking remain limited, and the effects of processing on the aroma of the resulting wines remain poorly understood. This study screened six edible rose cultivars for pigment characteristics, bioactive compounds, antioxidant capacity, and sugar composition, and the best-performing cultivar, *Rosa rugosa* ‘Dianhong’, was used to optimize two types of rose wine: a honey-fermented wine and a macerated wine. Among the six cultivars evaluated, *Rosa rugosa* ‘Dianhong’ had the highest anthocyanin and flavonoid contents and the strongest antioxidant capacity, and its sugar content was comparable to that of *Rosa centifolia.* For the honey-fermented wine, the best results were obtained with 20% initial sugar, 0.30 g/L yeast inoculum, and fermentation at 25 °C, producing a wine with 10.1% (*v*/*v*) alcohol with a harmonious floral–wine aroma profile and a typical rose-red color. For the macerated wine, a material-to-solvent ratio of 1:20 (*w*/*v*), 40% *v*/*v* base alcohol, and 60 days of maceration gave an orange-red wine with a mellow palate and a distinct rose aroma. SPME-GC×GC-TOF-MS identified 272 volatile compounds in the petals and wines; esters and alcohols were the most abundant chemical classes in both wine types, but fermentation and maceration shaped the volatile profiles in different ways. These results provide a practical reference for the selection of raw materials and the processing of rose-derived products.

## 1. Introduction

Edible roses are deciduous shrubs belonging to the genus Rosa (Rosaceae), encompassing original species, variants, and hybrid cultivars. These species exhibit extensive morphological diversity, intense floral aromas, and a wide spectrum of flower colors [1]. Edible roses contain significantly higher levels of nutrients than common fruits and vegetables and are rich in bioactive compounds (polysaccharides, polyphenols, and flavonoids) and bioavailable minerals [2]. Edible roses have been reported to enhance immunity, improve skin condition and regulate endocrine function; they also help prevent cardiovascular diseases and diabetes and inhibit cancer-cell proliferation and melanin secretion. Consequently, edible roses possess substantial application value in the food and pharmaceutical industries [3,4,5].

For rose-based beverage production, cultivar choice determines the concentrations of fermentable sugars, polyphenols, and pigments that will be carried into the final product, and antioxidant capacity is a key index of both functional value and processing stability, with polyphenols and flavonoids serving as the primary antioxidants in rose petals. Previous studies revealed that *Rosa rugosa* Thunb. f. exhibits the strongest antioxidant activity among edible rose cultivars grown in Yunnan [6]. Total flavonoids and total polysaccharides extracted from the Hotan rose display varying degrees of free radical scavenging capacity [7]. Among various roses cultivated in Beijing, the Miaofengshan rose possesses the optimal total flavonoid-mediated radical scavenging ability [8,9]. However, these evaluations rarely connected raw-material traits to downstream winemaking performance. Among various value-added products, rose wine stands out as a popular health beverage that largely retains the floral characteristics and antioxidant components of the raw petals.

Numerous studies have optimized processing parameters for fermented rose wine, focusing on fermentation temperature, material-to-solvent ratio, initial sugar content, yeast inoculum size, and fermentation duration [10,11]. Fermented wine prepared from *Rosa rugosa* var. plena has been traditionally consumed in certain regions. Alternative fermentation protocols utilizing sucrose, honey, or mixed fruits can produce composite flower-fruit wines with diversified flavor profiles [12,13,14,15]. Research on macerated rose wine (the macerated product is an ethanol-infusion-based beverage, legally classified as a flavored alcoholic beverage, lujiu (GB/T 27588-2011) in China [16]; this term is adopted hereafter in line with the established literature) remains comparatively limited. Both fresh and dried rose petals can serve as raw materials, with maceration duration, base-alcohol concentration, and material-to-solvent ratio being key determinants of final wine quality [17,18,19]. Macerated rose wine has been traditionally associated with cardiovascular benefits. Commercial products, including Luzhou Laojiao rose wine, rose Fen liquor, and rose liqueur, each present unique sensory characteristics with favorable taste and health-promoting effects [20,21,22,23]. The two categories are, however, fundamentally different products: honey-fermented rose wine is a low-alcohol wine produced by yeast fermentation of honey-derived sugars, whereas macerated rose wine is a spirit-based beverage in which ethanol acts as the extraction solvent for petal constituents; they follow distinct quality formation routes and are therefore evaluated separately in this study.

This study addressed two practical questions: which edible rose cultivar is the most suitable raw material for winemaking, and how the two types of rose wine should be processed to best preserve their quality and aroma. Although China has abundant cultivated edible rose germplasm, few studies have compared fermented and macerated rose wines prepared from the same raw material under identical conditions, and it is still unclear how fresh and dried petals differ in the volatile profiles of the resulting wines. Therefore, cultivar screening, process optimization, and aroma analysis were integrated into a single workflow. First, six mainstream edible rose cultivars in China were compared, with emphasis on traits most relevant to winemaking: fermentable sugars, polyphenolic profiles, pigments, and antioxidant capacity. The selected cultivar was then used to produce a honey-fermented wine and a macerated wine, and single-factor and orthogonal experiments were used to evaluate the processing conditions of each type. Finally, SPME-GC×GC-TOF-MS was used to characterize the volatile profiles of the petals and the optimized wines, so that raw-material traits, processing conditions, and aroma composition could be examined within one study. This approach improves the utilization of rose raw materials and provides technical support for the standardized production of rose-derived wines.

## 2. Materials and Methods

### 2.1. Experimental Materials

Six two-year-old edible rose cultivars, including *Rosa rugosa* ‘Chongban’, *Rosa rugosa* ‘Ji’, *Rosa rugosa* ‘Dianhong’, *Rosa centifolia*, *Rosa damascena,* and *Rosa rugosa* ‘Pingyin’, were cultivated under uniform management conditions at the Wandong Comprehensive Experimental Station of Anhui Agricultural University (Mingguang, Anhui Province, China; 32°46′ N, 117°58′ E). Plants were grown in sandy loam soil under open-field conditions with standard drip irrigation, annual pruning in early spring, and routine pest management. Flowers were harvested at the fully open stage between 6:00 and 8:00 a.m. on cloudless days, and petals were used within 2 h of harvest.

The fermentation strain was *Saccharomyces cerevisiae* (RW yeast, specially developed for fruit wine fermentation; Angel Yeast Co., Ltd., Yichang, China). Pectinase: Lafase He Grand Cru, enzymatic activity of 18,000 U/g, Laffort, Bordeaux, France. Food-grade honey was purchased from Haohao Apiculture Co., Ltd., Mingguang City, China. The base liquor was purchased from Jingtai Rose Technology Co., Ltd. (Hefei, China).

### 2.2. Preparation Methods of Dianhong Rose Wine

#### 2.2.1. Honey-Fermented Rose Wine

Honey-fermented *Rosa rugosa* Dianhong wine was prepared as follows. Distilled water was used for honey dilution to avoid residual chlorine, mineral ions and heavy metals that may inhibit yeast activity or produce off-flavors. It was boiled, cooled to 35 °C and mixed with honey to sterilize the water, remove dissolved oxygen, protect thermolabile components in honey and maintain proper temperature for yeast activation. The initial sugar content was adjusted to the target level using a handheld refractometer (PAL-1, Atago, Tokyo, Japan) and expressed as °Brix. Fresh petals of *Rosa rugosa* ‘Dianhong’, harvested as described in Section 2.1, were sorted, washed three times with distilled water, drained, and added to the cooled honey solution at a petal-to-solution ratio of 1:20 (*w*/*v*). *Saccharomyces cerevisiae* (Angel yeast, Angel Yeast Co., Ltd., Yichang, China) was activated in sterile water at a 1:10 (*w*/*v*) ratio at 35 °C for 10 min. The activated yeast, pectinase (0.02 g/L), and citric acid (1.50 g/L, to adjust initial pH to ~3.5) were added to the honey solution and mixed thoroughly in 1 L glass fermentation bottles equipped with airlocks. Fermentation was conducted at 25 °C in the dark and terminated when reducing sugar content dropped below 5 g/L.

Single-factor experiments were carried out with three replicates for each group, while other conditions were kept constant. Based on extensive preliminary experiments, the experimental gradients were set as follows: initial sugar content (16, 18, 20, 22, 24% (*w*/*v*)), yeast inoculation dosage (0.10, 0.20, 0.30, 0.40, 0.50 g/L), and fermentation temperature (20, 22.5, 25, 27.5, 30 °C). After one month of aging, physicochemical properties, color parameters, antioxidant indices, and sensory scores served as response variables to investigate the effects of initial sugar content, yeast inoculation dosage and fermentation temperature on the quality of Dianhong rose fermented wine, to identify optimal levels of each fermentation factor.

#### 2.2.2. Macerated Rose Wine

The ‘Dianhong’ macerated rose wine was prepared as follows: Intact fresh rose petals free from rot and mechanical damage, and dried rose flowers were selected as raw materials. Fresh petals were freeze-dried at −80 °C for 24 h until constant weight. In all comparisons between fresh-petal and freeze-dried petal-macerated wines, the two raw materials were used on an equal fresh-mass basis. The solid-to-solvent ratio of rose materials (fresh/dried flowers) to base liquor was set at 1:20 (*w*/*v*), and the alcohol content of the base liquor was 55% (*v*/*v*). The mixture was sealed in bottles and kept in a constant-temperature incubator at 20 °C for 60 days of maceration. After maceration, the mixture was filtered, pasteurized at 87 °C for 20 min, cooled to room temperature and bottled. Low-alcohol macerated wine was prepared by alcohol dilution according to the formula: Base liquor volume × Base liquor alcohol content = Diluted wine volume × Diluted wine alcohol content.

Single-factor experiments were performed with three parallel replicates per group under fixed auxiliary conditions. Based on extensive preliminary experiments, the tested gradients included solid-to-solvent ratios (1:10, 1:20, 1:30, 1:40, 1:50), maceration duration (20, 40, 60, 80, 100 d), and base liquor alcohol content (35, 40, 45, 50, 55% *v*/*v*). After one month of storage, physicochemical characteristics, color, antioxidant capacity and sensory evaluation scores were used as comprehensive indicators to analyze the influences of solid-to-solvent ratio, maceration time and base liquor alcohol content on the quality of ‘Dianhong’ macerated rose wine, and the optimal level of each maceration parameter was determined.

### 2.3. Determination Methods of Indicators

#### 2.3.1. Physicochemical Indicators

The physicochemical indicators were determined as follows. pH was measured with a pH meter calibrated with standard buffer solutions (pH 4.01 and 7.00) before each session; soluble solids content was determined with a handheld refractometer (PAL-1, Atago, Tokyo, Japan) and expressed as °Brix; total sugar and reducing sugar contents were quantified by Fehling’s titration according to GB/T 15038-2006 (General analytical methods of wine and fruit wine); total acids were determined by acid–base titration and calculated on the basis of acetic acid; and alcohol content was measured with the pycnometer method according to GB/T 15038-2006 [24].

#### 2.3.2. Color Measurement

Color parameters (L*, a*, b*) of petal extracts and wine samples were determined with a CR-400/410 color difference meter (Konica Minolta, Tokyo, Japan). The instrument was calibrated against the supplied standard white calibration plate before each measurement session, and measurements were performed with the D65 illuminant and a 10° observer angle. Petal extracts were prepared by extracting 1.0 g of petals with 10 mL of 50% (*v*/*v*) ethanol for 24 h at room temperature in the dark; extracts and wine samples were placed in transparent glass cuvettes, and three independent replicates were measured for each sample. The total color difference (Δ*E*) was calculated as Δ*E* = √[(ΔL*)^2^ + (Δa*)^2^ + (Δb*)^2^] [25].

#### 2.3.3. Antioxidant-Related Indicators

Total phenolic content was measured using the Folin–Ciocalteu colorimetric method and expressed as gallic acid equivalents [26]. Total flavonoid content was determined by the aluminum nitrate colorimetric method and expressed as rutin equivalents [27]. Total anthocyanin content was quantified by the pH differential method (pH 1.0 and pH 4.5 buffers) and expressed as cyanidin-3-glucoside equivalents [28].

#### 2.3.4. Sensory Evaluation

The sensory evaluation form was established with slight modifications based on the Chinese national standard GB/T 27588-2011 for liquor [16] and the flavor profile test GB/T 12313-1990 [29]. A panel of ten trained assessors (staff and graduate students of the College of Food and Nutrition, Anhui Agricultural University) with prior experience in fruit-wine sensory evaluation was recruited. The panelists were trained with reference standards representing the evaluated attributes (rose floral aroma, honey sweetness, and wine aroma) before formal scoring, and the aroma intensity of the six edible rose cultivars was evaluated independently by the panel. Wine samples were evaluated in individual sensory booths under neutral lighting; 30 mL of each sample was served at room temperature in opaque glasses coded with three-digit random numbers, and mineral water was provided for palate cleansing. Appearance (20 points), aroma (30 points), taste (40 points), and overall style (10 points) were scored, giving a total of 100 points (Table A1). Each assessor scored every sample once, and the reported scores are the means of the three independent biological replicates ± standard deviation across the panel. One-way ANOVA was applied to the panel scores to detect significant differences among treatments, and panel consistency was monitored using replicate reference samples.

#### 2.3.5. Volatile Aroma Compounds Analysis

Volatile aroma compounds were analyzed by SPME-GC×GC-TOF-MS using a LECO Pegasus BT 4D system equipped with an Agilent 8890A GC, a dual-stage jet modulator, and a split/splitless inlet. A dual-column set was used: Rxi-5Sil MS (30 m × 250 μm × 0.25 μm) as the primary column and Rxi-17Sil MS (2 m × 150 μm × 0.15 μm) as the secondary column. For SPME, a 1 mL sample was placed in a 20 mL headspace vial containing 4 mL saturated NaCl solution and 10 μL internal standard (2-octanol, 100 mg/L), then extracted with a DVB/CAR/PDMS fiber (50/30 μm) at 40 °C for 30 min. GC conditions: helium carrier gas at 1.0 mL/min; primary oven: 40 °C (3 min) → 200 °C at 4 °C/min → 250 °C at 10 °C/min (5 min); secondary column temperature offset +5 °C; modulator temperature offset +15 °C; modulation period 6 s. Compound identification was performed by matching mass spectra against the NIST 2020 library (match factor ≥ 800), combined with retention index (RI) verification using a C8–C20 n-alkane series. Identification was considered positive when the RI deviation was ≤20 units relative to literature values [30,31]. As no authentic standards were used, all compounds are reported as tentatively identified.

### 2.4. Data Processing and Analysis

All experiments were conducted with three independent biological replicates (independent fermentation or maceration batches, or independent petal samples collected from different plants), and each analytical determination was performed in triplicate (technical replicates); data are presented as mean ± standard deviation (SD). Raw data were processed using Microsoft Excel 2022 (Microsoft Corp., Redmond, WA, USA). Before parametric testing, the normality and the homogeneity of variance were verified with the Shapiro–Wilk test and Levene’s test, respectively; one-way ANOVA combined with Tukey’s honestly significant difference (HSD) test was employed for multiple comparisons, with significance set at *p* < 0.05, and exact *p* values are reported in the text where applicable. The comprehensive score of the orthogonal fermentation test was calculated as follows. The quality indices of each treatment—the physicochemical indices, the color index, the antioxidant index, and the sensory total score—were each normalized to a value ranging from 0 to 1 using the min–max normalization method. Pearson correlation analysis was performed to evaluate relationships among variables. OPLS-DA was performed with SIMCA 14.1 (Sartorius Stedim Data Analytics, Umeå Sweden) using unit-variance scaling; model robustness was assessed by seven-fold and a 200-time permutation test, and compounds with a variable importance in projection (VIP) > 1 and *p* < 0.05 were regarded as differential volatile compounds.

## 3. Results

### 3.1. Comprehensive Quality Analysis of Six Edible Rose Cultivars

#### 3.1.1. Analysis of Appearance and Aroma Intensity of Different Edible Rose Cultivars

**Figure 1:**
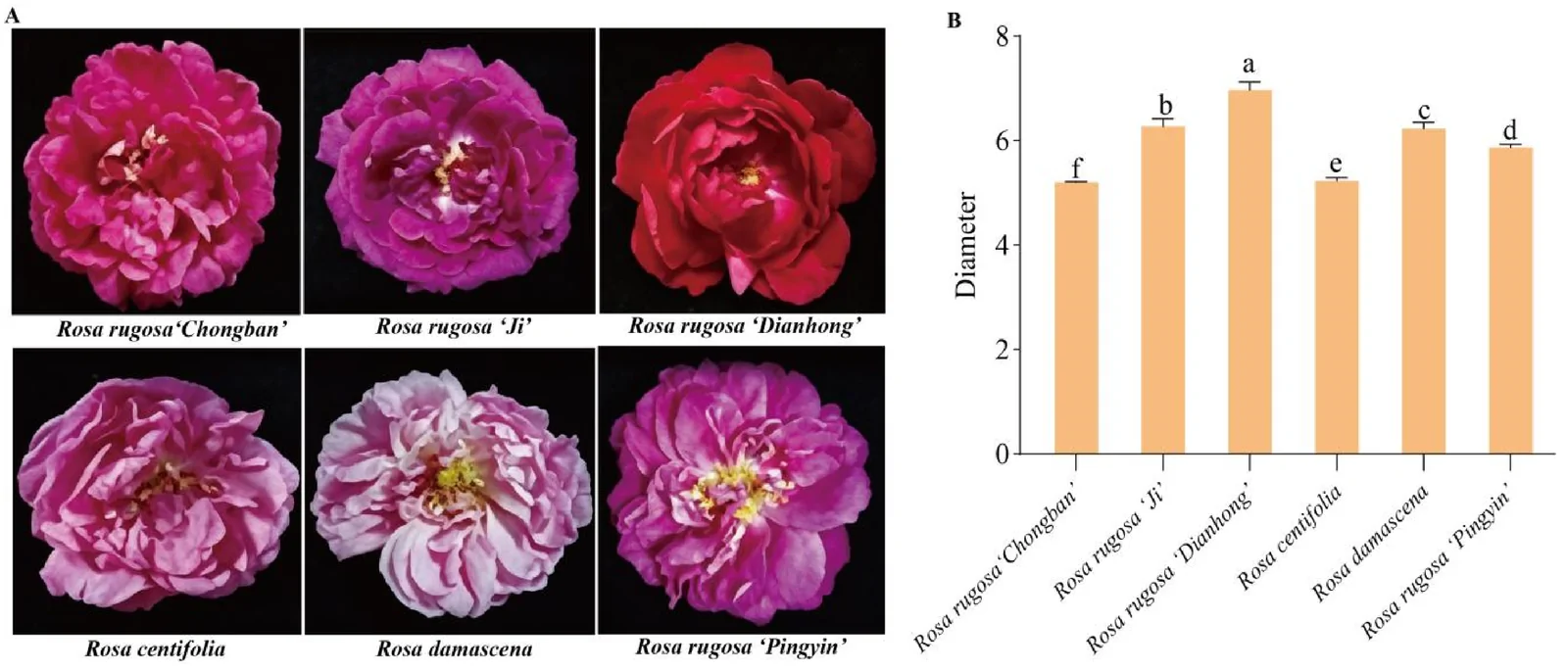
Floral phenotypes and corolla diameter comparison of six edible rose cultivars. (**A**) Floral morphology of six edible rose cultivars; (**B**) Comparison of corolla diameter of six edible rose cultivars. Different lowercase letters on the top of bars denote significant differences at *p* < 0.05.

The morphological phenotypes of the six cultivars are shown in Figure 1A. Among them, *Rosa rugosa* ‘Dianhong’ had the largest corolla diameter and bright crimson petals (Figure 1B). The floral aroma intensity of the six cultivars was ranked as follows: *Rosa rugosa* ‘Dianhong’ (1) > *Rosa rugosa* ‘Pingyin’ (2) > *Rosa damascena* (3) > *Rosa centifolia* (4) > *Rosa rugosa* ‘Ji’ (5) > *Rosa rugosa* ‘Chongban’ (6). *Rosa rugosa* ‘Chongban’, ‘Ji’, and ‘Pingyin’ exhibited bright coloration and are thus suitable for food processing, whereas *Rosa centifolia* and *Rosa damascena*—despite their pale petals—were preferred for fragrance extraction in cosmetics.

#### 3.1.2. Comprehensive Analysis of Physicochemical and Antioxidant Indexes of Different Edible Rose Cultivars

**Figure 2:**
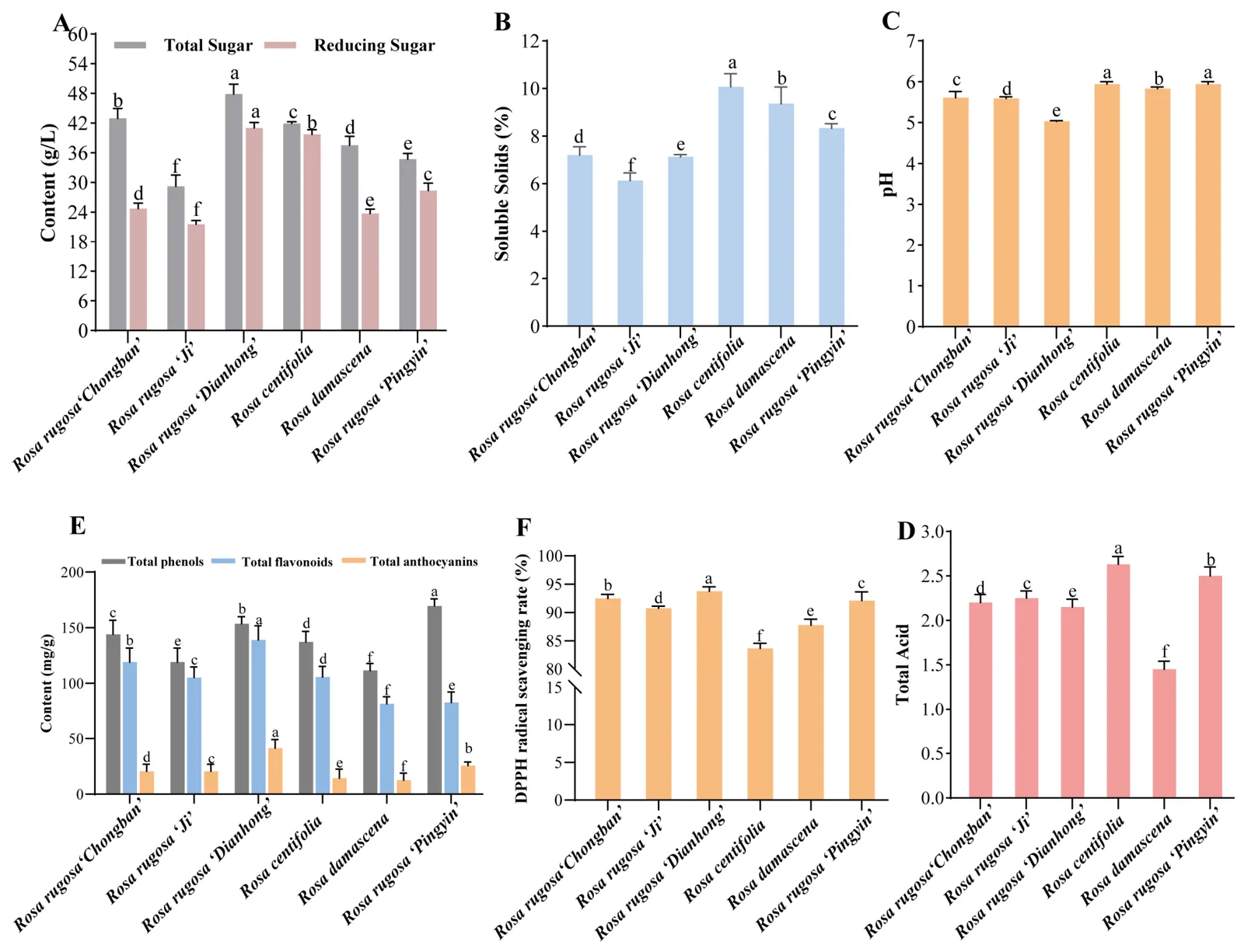
Basic physicochemical and antioxidant indexes of petals from six edible rose cultivars. (**A**) Total sugar and reducing sugar content; (**B**) Soluble solids content; (**C**) pH value; (**D**) Total acid content; (**E**) Total phenols, total flavonoids and total anthocyanins content; (**F**) DPPH radical scavenging rate. Different lowercase letters above each bar indicate significant differences among cultivars at *p* < 0.05.

The basic physicochemical parameters are summarized in Figure 2. Significant differences (*p* < 0.05) in total sugar and reducing sugar contents were detected among the six rose cultivars. *Rosa rugosa* ‘Dianhong’ and *Rosa centifolia* accumulated the highest levels of total sugar and reducing sugar (Figure 2A), identifying them—among the cultivars evaluated—as the most promising raw materials for fermented rose wine production. Soluble solids content also differed significantly (*p* < 0.05) across cultivars, ranging from 6.13% to 10.07%, with *Rosa centifolia* showing the maximum value (Figure 2B). The pH (Figure 2C) and total acids (Figure 2D) of petals from the six cultivars varied within a narrow range, and all samples exhibited weak acidity overall. As illustrated in Figure 2E, the total phenolic contents of *Rosa rugosa* ‘Pingyin’ and *Rosa rugosa* ‘Dianhong’ were significantly higher than those of the other cultivars, whereas *Rosa damascena* had the lowest total phenol content. *Rosa rugosa* ‘Dianhong’ possessed the highest contents of total flavonoids and total anthocyanins (*p* < 0.05), whereas *Rosa damascena* contained the lowest. All six cultivars exhibited strong DPPH radical scavenging activity with scavenging rates above 50%, and *Rosa rugosa* ‘Dianhong’ showed significantly stronger (*p* < 0.05) overall antioxidant capacity than the other cultivars (Figure 2F).

#### 3.1.3. Analysis of Color Parameters of Petal Extracts from Different Edible Rose Cultivars

**Figure 3:**
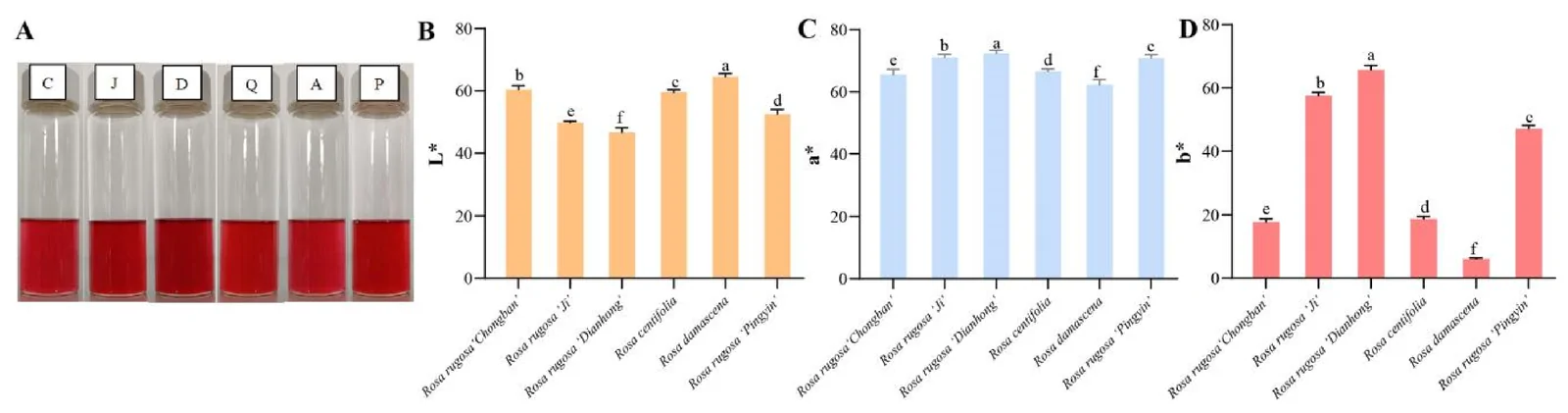
Visual appearance and color parameters of ethanol extracts from six edible rose cultivars. (**A**) Visual appearance of ethanol extracts of six rose cultivars; (**B**) Lightness (L*) value; (**C**) Red-green (a*) value; (**D**) Yellow-blue (b*) value. C, J, D, Q, A and P represent *Rosa rugosa* ‘Chongban’, *Rosa rugosa* ‘Ji’, *Rosa rugosa* ‘Dianhong’, *Rosa centifolia*, *Rosa damascena*, and *Rosa rugosa* ‘Pingyin’, respectively. Different lowercase letters above each bar indicate significant differences among cultivars at *p* < 0.05.

The L*, a*, and b* color coordinates of ethanol extracts from six edible rose cultivars, representing lightness, red-green chromaticity, and yellow-blue chromaticity, are shown in Figure 3A. As shown in Figure 3C, the extract of *Rosa rugosa* ‘Dianhong’ exhibited the highest a* value, which was significantly higher than that of all other cultivars (*p* < 0.05), followed by *Rosa rugosa* ‘Ji’ and *Rosa rugosa* ‘Pingyin’, while *Rosa damascena* exhibited the lowest a* value. This trend in color parameter variation was consistent with the visual appearance of extracts in Figure 3A. Further analysis of L* and b* values indicated that the extract of *Rosa damascena* had the maximum L* value (Figure 3B), and the extract of *Rosa rugosa* ‘Dianhong’ showed the highest b* value (Figure 3D). All extracts displayed bright red hues, demonstrating great potential as natural red colorants.

#### 3.1.4. Correlation and Cluster Analysis of Indexes of Different Edible Rose Cultivars

**Figure 4:**
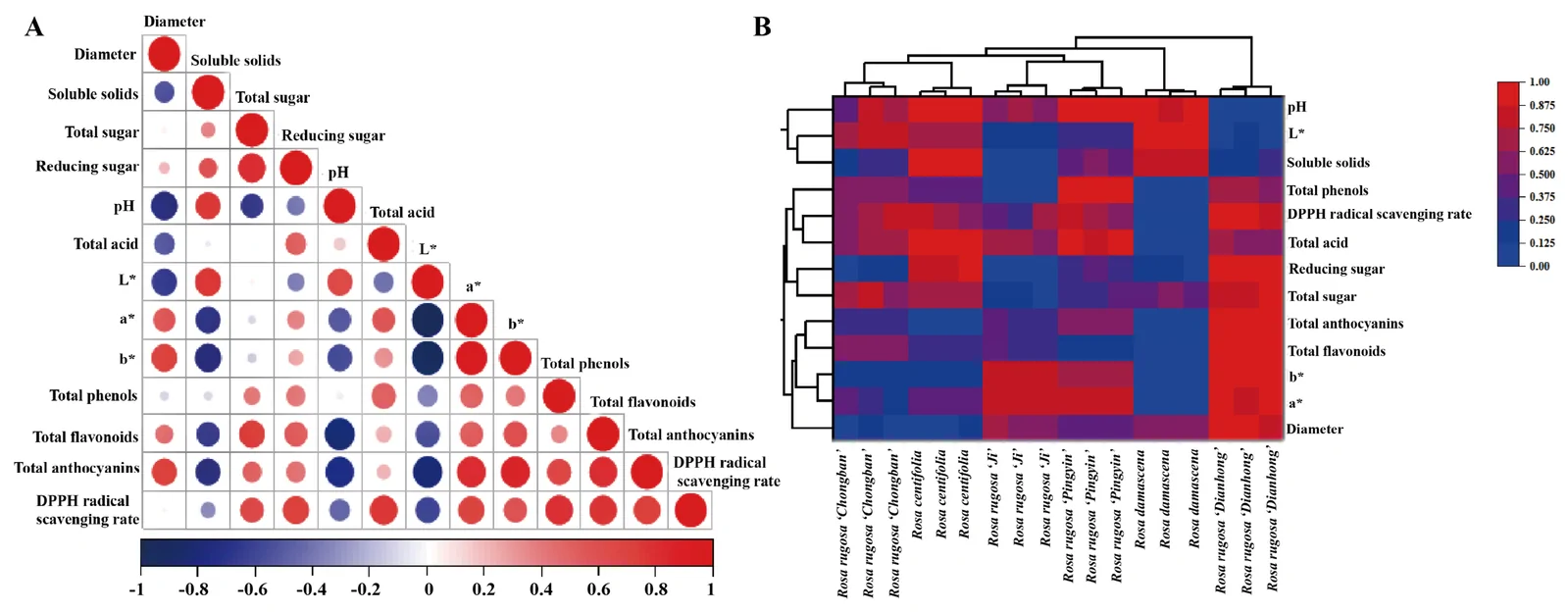
Correlation heatmap and cluster analysis of various indicators for six edible rose cultivars. (**A**) Correlation matrix heatmap of all measured indicators; (**B**) Cultivar clustering heatmap constructed based on all physicochemical and bioactive indicators.

The correlation analysis of each index is presented in Figure 4A. The DPPH radical scavenging rate showed significant positive correlations with anthocyanin content, total flavonoid content, total phenolic content, redness value a*, yellowness value b*, total acid content, and reducing sugar content. The pH value was significantly negatively correlated with total flavonoid content, anthocyanin content, and DPPH radical scavenging rate. Cluster analysis results (Figure 4B) showed that *Rosa rugosa* ‘Dianhong’ formed an independent cluster. This cultivar accumulated abundant bioactive compounds including total phenols, total flavonoids, and anthocyanins, together with high levels of total sugar and reducing sugar, rendering it suitable for the development of antioxidant-rich functional foods and rose wine products. Combined with the results of the correlation and cluster analyses, *Rosa rugosa* ‘Dianhong’ exhibited the most favorable comprehensive physicochemical quality and antioxidant activity among the six cultivars evaluated under the conditions tested, and was therefore prioritized for beverage development.

### 3.2. Process Optimization of Fermented Honey Wine with Rosa rugosa ‘Dianhong’ Petals

#### 3.2.1. Effects of Fermentation Conditions on Reducing Sugar Consumption in *Rosa rugosa* ‘Dianhong’ Fermented Honey Wine

**Figure 5:**
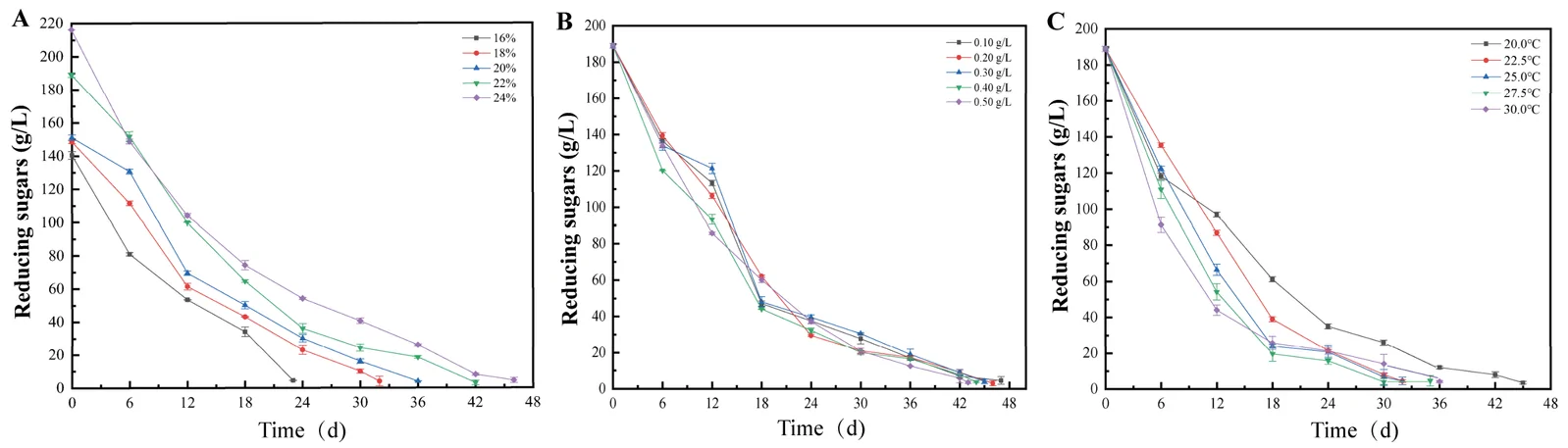
Dynamic changes in reducing sugar content in fermented honey wine of *Rosa rugosa* ‘Dianhong’ under different fermentation conditions. (**A**) Different initial sugar concentrations; (**B**) different yeast inoculation dosages; (**C**) different fermentation temperatures. Error bars represent the standard deviation of three biological replicates (*n* = 3).

As presented in Figure 5A, reducing sugar concentrations decreased continuously with yeast proliferation. Among treatments with different initial sugar contents, the 16% and 24% groups exhibited the fastest sugar consumption during days 0 to 6. The maximum sugar consumption rate of the 18% and 20% groups occurred from days 6 to 12, whereas the peak of the 22% group was delayed to days 12 to 24. Fermentation was completed on days 23, 32, 36, 42 and 46 for each sugar gradient, with residual reducing sugars lower than 5 g/L at the end of fermentation. In the yeast inoculation experiment (Figure 5B), rapid depletion of reducing sugars was observed in all groups within the first six days. The 0.50 g/L group showed the greatest reduction in sugar content during days 6 to 12. The 0.10, 0.40 and 0.50 g/L groups reached their maximum sugar consumption rates at days 12 to 18, while the peak sugar consumption of the 0.20 g/L group was postponed to days 18 to 24. All inoculation treatments finished fermentation within 43 to 47 days, and the 0.50 g/L group reached the fermentation endpoint first. For the temperature gradient trials (Figure 5C), the 30 °C group consumed sugars rapidly from days 0 to 6. Active sugar consumption of the 22.5, 25 and 27.5 °C groups took place between days 6 and 18, and the 20 °C group maintained the slowest sugar utilization rate throughout the fermentation process. The 22.5 °C and 25 °C groups completed fermentation first, and the 20 °C group required the longest fermentation period.

The fermentation-rate differences observed among treatments had direct quality implications. Treatments that consumed sugar rapidly (the 24% initial-sugar group during days 0 to 6, the 0.50 g/L inoculum group, and the 30 °C group) generally completed fermentation earlier but showed lower retention of total phenols and total flavonoids and lower sensory scores in the finished wines (Table 1 and Table 2), indicating a trade-off between fermentation speed and the accumulation of quality-related metabolites. Conversely, excessively slow fermentation (16% initial sugar, 0.10 g/L inoculum, 20 °C) prolonged the process without improving quality retention. Intermediate conditions (18–22% initial sugar, 0.20–0.30 g/L inoculum, 22.5–27.5 °C) balanced fermentation efficiency with phenolic retention and sensory quality and were selected for subsequent orthogonal optimization.

#### 3.2.2. Effects of Fermentation Conditions on Quality Characteristics of Fermented Honey Wine of *Rosa rugosa* ‘Dianhong’

As shown in Table 1, the 24% initial sugar group exhibited the highest L* value (maximum lightness), whereas the 16% group showed a lower L* value but a higher a* value. Increasing the initial sugar concentration inhibited the dissolution of bioactive compounds from the rose petals but improved the brightness of the wine, and the soluble solids content increased continuously with sugar concentration. Notably, the wine fermented with 20% initial sugar had the highest contents of total phenols and total flavonoids. Yeast inoculation dosage significantly affected the color of the wine: the 0.30 g/L group reached a peak a* value of 15.21, and with increasing inoculum size the contents of total phenols and total flavonoids first increased and then decreased. A high inoculation dosage accelerated fermentation and shortened the fermentation cycle, but 0.30 g/L was the optimal dosage for the accumulation of phenolic antioxidants. Fermentation temperature also regulated the color of the wine. The 25 °C group had the lowest L* value and a maximum a* value of 30.91; both the 25 °C and 27.5 °C treatments gave the maximum total flavonoid content of 6.23 mg/g, with similar total phenol contents. Temperatures above 27.5 °C, however, induced the degradation of phenolic and flavonoid compounds and reduced their final accumulation in the wine.

#### 3.2.3. Sensory Evaluation of Fermented Honey Wine of *Rosa rugosa* ‘Dianhong’ Under Different Fermentation Conditions

**Figure 6:**
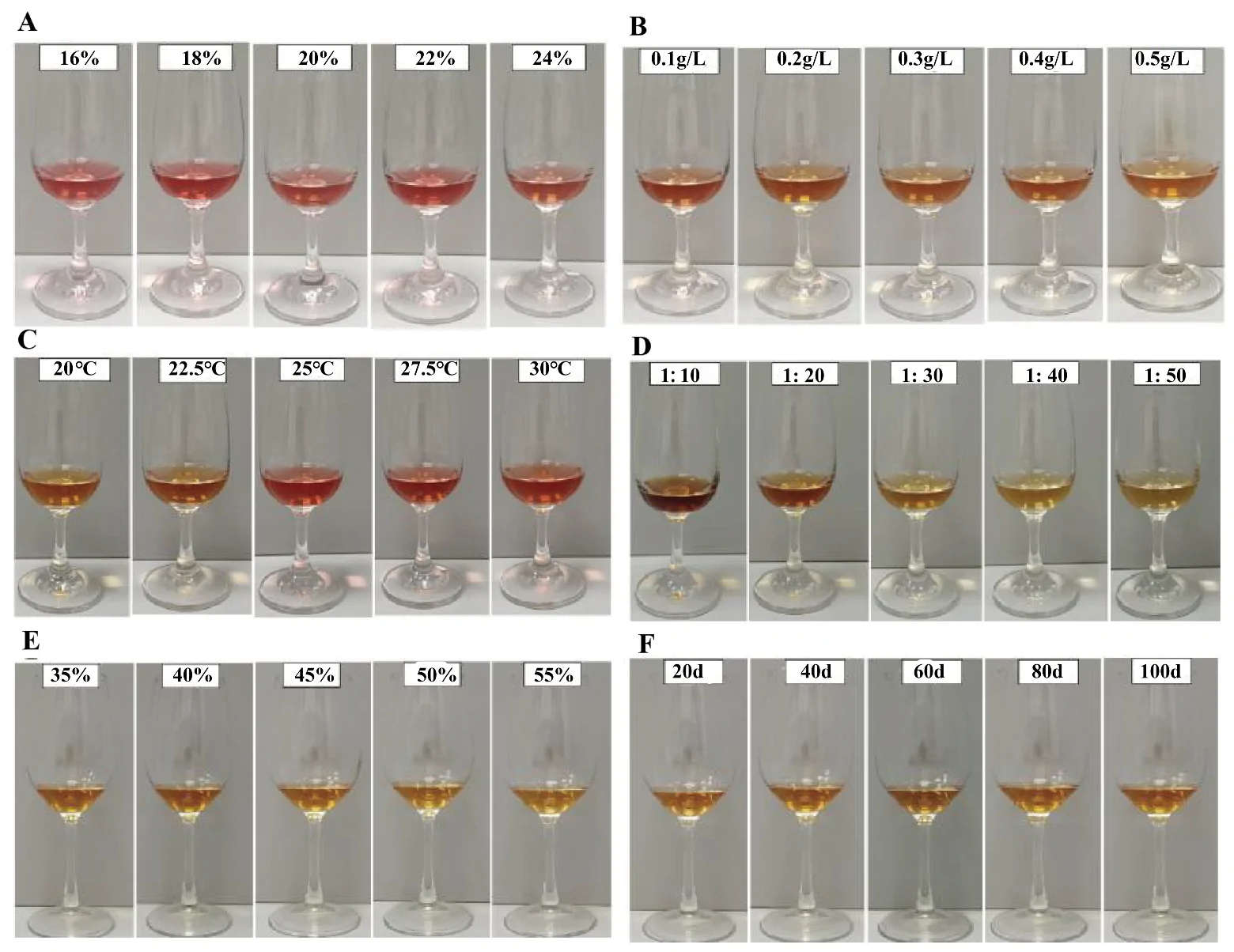
Visual appearance of ‘Dianhong’ rose honey-fermented wine (**A**–**C**) and rose macerated wine (**D**–**F**) treated with different processing conditions. (**A**) Initial sugar levels; (**B**) yeast addition amounts; (**C**) fermentation temperatures; (**D**) solid-to-solvent ratios; (**E**) alcohol concentrations of base wine; and (**F**) maceration time.

Ten panelists evaluated honey-fermented wine prepared with various initial sugar concentrations according to the sensory scoring criteria in Appendix A. The results are summarized in Table 2. Wines fermented at 16% initial sugar concentration received high scores for color and clarity. For aroma attributes (Figure 6A), the 16% and 18% sugar treatments produced intense and pure aromatic profiles. In terms of taste, the wine produced at 20% initial sugar concentration achieved superior scores owing to its full-bodied, well-balanced taste with prominent honey and rose floral notes. Significant differences were detected in overall wine style. Specifically, the sample fermented at 20% sugar possessed the typical style of rose wine and achieved the highest comprehensive sensory score. Significant differences in color and clarity were also found among wines fermented with different yeast inoculation dosages (Figure 6B). The 0.20 g/L inoculation group received high scores for color, exhibiting a clear, glossy appearance. Wines fermented at 0.20 g/L and 0.30 g/L yeast dosages exhibited rich, clean aromas. Regarding taste, wines fermented with 0.20 g/L and 0.30 g/L yielded full-bodied and harmonious wines with strong honey flavor, and the 0.30 g/L inoculation dosage achieved the maximum total sensory score. Fermentation temperature significantly affected the visual appearance of the finished wine (Figure 6C). Samples fermented at 20 °C and 22.5 °C received low color scores but exhibited high clarity. For aroma attributes, wines fermented at 25 °C exhibited an intense rose aroma and obtained the highest aroma scores.

#### 3.2.4. Orthogonal Test of the Fermentation Technology for *Rosa rugosa* ‘Dianhong’ Fermented Honey Wine

Based on the results of single-factor experiments, three critical influencing factors including initial sugar concentration (18%, 20%, 22%), yeast inoculation dosage (0.2 g/L, 0.3 g/L, 0.4 g/L) and fermentation temperature (22.5 °C, 25 °C, 27.5 °C) were selected. A three-factor and three-level orthogonal array L_9_(3^4^) was adopted to carry out the orthogonal experiment.

**Figure 7:**
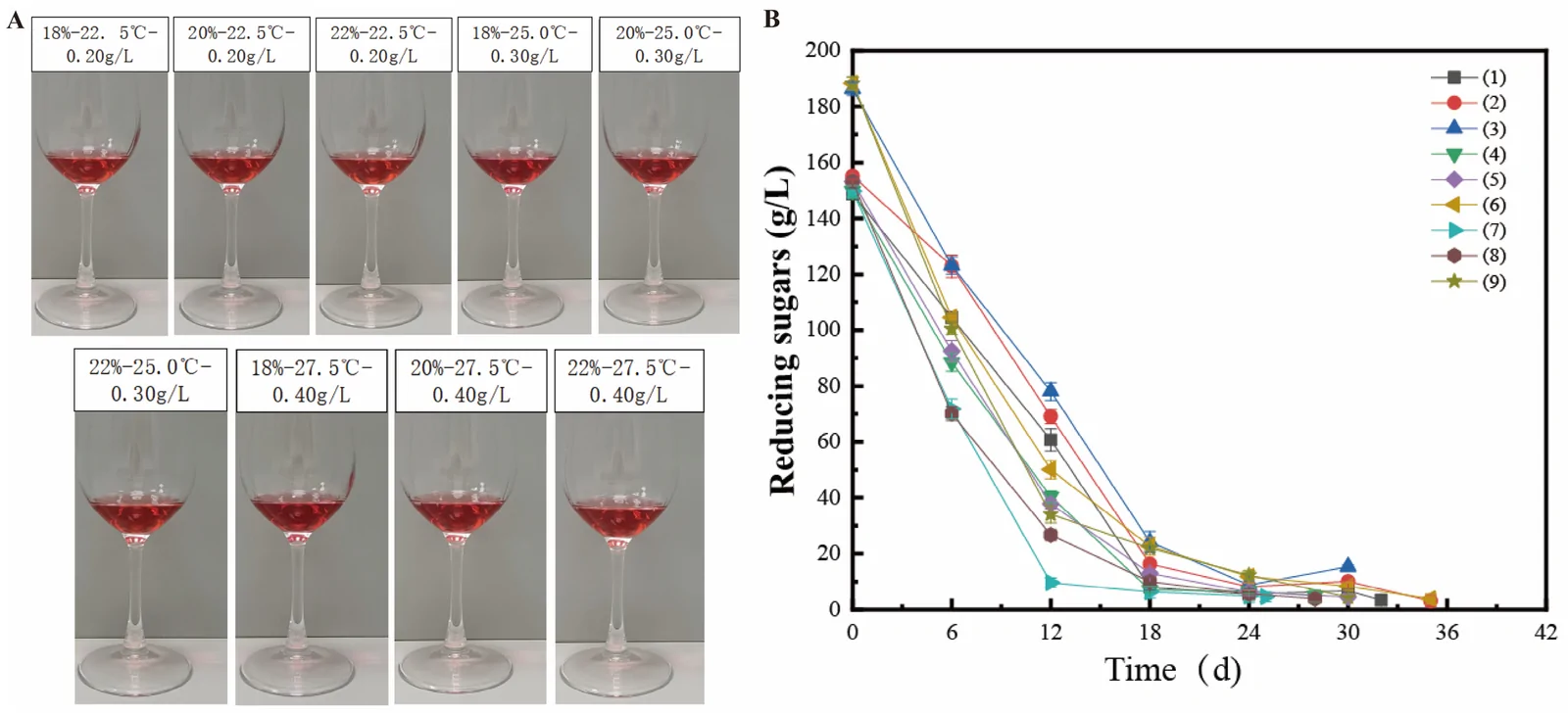
Visual appearance (**A**) and dynamic variations (**B**) in reducing sugar content of fermented honey wine of *Rosa rugosa* ‘Dianhong’ under nine orthogonal fermentation treatments. Numbers (1)–(9) correspond to the nine orthogonal test treatments listed in Table 3. Error bars represent the standard deviation of three biological replicates (*n* = 3).

Dynamic changes in reducing sugar content under different fermentation conditions are presented in Figure 7A. The reducing sugar content of all treatment groups continuously decreased along with yeast proliferation. Treatment 7 (18% initial sugar concentration, 0.4 g/L yeast inoculation dosage, 27.5 °C fermentation temperature) showed the fastest reduction in reducing sugar during days 0 to 12 of fermentation, and the rate of decrease gradually slowed down in the later stage. The remaining eight treatments reached their peak sugar consumption within 0 to 18 days. Treatment 7 achieved fermentation completion earliest, while Treatment 2 and Treatment 6 had the longest fermentation durations. The overall fermentation period of the nine groups ranged from 25 to 35 days.

The visual appearance of fermented *Rosa rugosa* ‘Dianhong’ honey wine under different orthogonal processes is shown in Figure 7B, and the corresponding quality indicators are summarized in Table 3. After one month of aging, the wine fermented under the conditions of 20% initial sugar concentration, 0.30 g/L yeast inoculation dosage, and 25 °C exhibited the best comprehensive quality. Its total phenolic content, total flavonoid content, and total color difference Δ*E* value reached 14.39 mg/g, 7.35 mg/g, and 68.36, respectively, with the highest sensory score of 77.65 among all treatments.

Results of the orthogonal fermentation test (Table 4) were analyzed by range analysis. The range analysis indicated the following order of factor significance: B > A > C, indicating the factor priority: yeast inoculation dosage > initial sugar concentration > fermentation temperature. The optimal combination was A2B2C2 (20% initial sugar concentration, 0.30 g/L yeast inoculation dosage, 25 °C fermentation temperature), which achieved the highest comprehensive score. Verification experiments under A2B2C2 conditions yielded the highest comprehensive score of 4.90 (on a 5-point scale), together with a sensory total score of 77.65 (on a 100-point scale, Table 3), reducing sugar of 9.87 g/L, total acids of 3.40 g/L (as acetic acid), total anthocyanin content of 6.83 mg/g, and DPPH radical scavenging activity of 53.63%.

### 3.3. Process Optimization of Rosa rugosa ‘Dianhong’ Macerated Wine

#### 3.3.1. Determination of Physicochemical Indicators Under Varied Solid-to-Solvent Ratios, Base Wine Alcohol Concentrations and Maceration Periods

The color parameters and physicochemical indices of *Rosa rugosa* ‘Dianhong’ macerated wine under different treatments are shown in Table 5. The wine sample prepared at a solid-to-solvent ratio of 1:10 possessed the highest a* value. As the solid-to-solvent ratio increased, the L* lightness value decreased from 88.55 to 59.89, whereas the a* redness increased gradually from 0.41 to 25.36, and the b* yellowness rose from 19.83 to 82.12. Meanwhile, the contents of total phenols, total flavonoids, and soluble solids of wine samples increased continuously. The 1:10 treatment group exhibited the highest accumulation of bioactive substances. With the increase in base wine alcohol concentration, the L* lightness of macerated wine increased slightly from 83.46 to 85.83. The a* value peaked at the alcohol concentration of 40% *v*/*v* and then decreased gradually, while the b* value declined continuously from 37.30 to 25.69. Higher total phenol contents were obtained under 40% *v*/*v* and 55% *v*/*v* conditions. The soluble solids content increased with the elevation of alcohol concentration, ranging from 12.40% to 16.87%. All *Rosa rugosa* ‘Dianhong’ macerated wine samples showed an orange-red hue. As the maceration time extended, the L* value slowly decreased from 85.07 to 82.88, accompanied by synchronous increases in a* and b* values. The contents of total phenols, total flavonoids, and soluble solids continuously accumulated with extended maceration time. However, antioxidant bioactive compounds degraded after 60 days of maceration, resulting in a significant decrease in total phenol and total flavonoid contents.

#### 3.3.2. Sensory Evaluation Under Varied Solid-to-Solvent Ratios, Base Wine Alcohol Concentrations and Maceration Periods

Single-factor sensory analysis (Table 6) identified the best maceration conditions within the tested ranges for *Rosa rugosa* ‘Dianhong’ macerated wine as a solid-to-solvent ratio of 1:20 (*w*/*v*), 40% (*v*/*v*) base alcohol, and 60-day maceration, which provided the optimal balance between overall sensory scores and bioactive constituent retention.

As the solid-to-solvent ratio increased from 1:10 to 1:50, wine redness decreased while clarity improved (Figure 6D). The 1:10 and 1:20 treatments exhibited an intense rose aroma, attributed to higher petal dosages releasing more aromatic compounds. The 1:20 sample achieved a fuller, more harmonious mouthfeel and typical rose wine attributes, obtaining the highest total score (69.4) among all ratio groups. Base alcohol concentration significantly affected sensory profiles. Wines macerated at 40% (*v*/*v*) showed significantly higher color scores than other treatments, with a transparent appearance free of suspended solids (Figure 6E). Treatments of 40–50% (*v*/*v*) exhibited better aroma coordination and mouthfeel fullness than the 35% (*v*/*v*) group, indicating that moderate alcohol strengths effectively retained native rose floral notes and harmonized the alcoholic aroma. The 40% (*v*/*v*) treatment achieved the peak comprehensive sensory score (70.50) with full-bodied texture and prominent rose wine features. By contrast, 55% (*v*/*v*) weakened rose floral characters, generated complex flavors and sharp pungency, and reduced sensory scores. Maceration duration also played a critical role. Extended maceration promoted anthocyanin leaching and deepened wine color (Figure 6F). The 60-day treatment reached the highest sensory score and darkest color with bright gloss. However, antioxidant degradation beyond 60 days faded wine color. Samples macerated for 60 and 100 days retained abundant genuine rose aroma with balanced floral and alcoholic notes. The 60-day group achieved significantly higher sensory scores than other durations, exhibiting intense rose fragrance and a prominent sweet flavor.

### 3.4. Quality Analysis of Honey Fermented Wine, Fresh Petal-Macerated Wine and Freeze-Dried Petal-Macerated Wine of Rosa rugosa ‘Dianhong’

**Figure 8:**
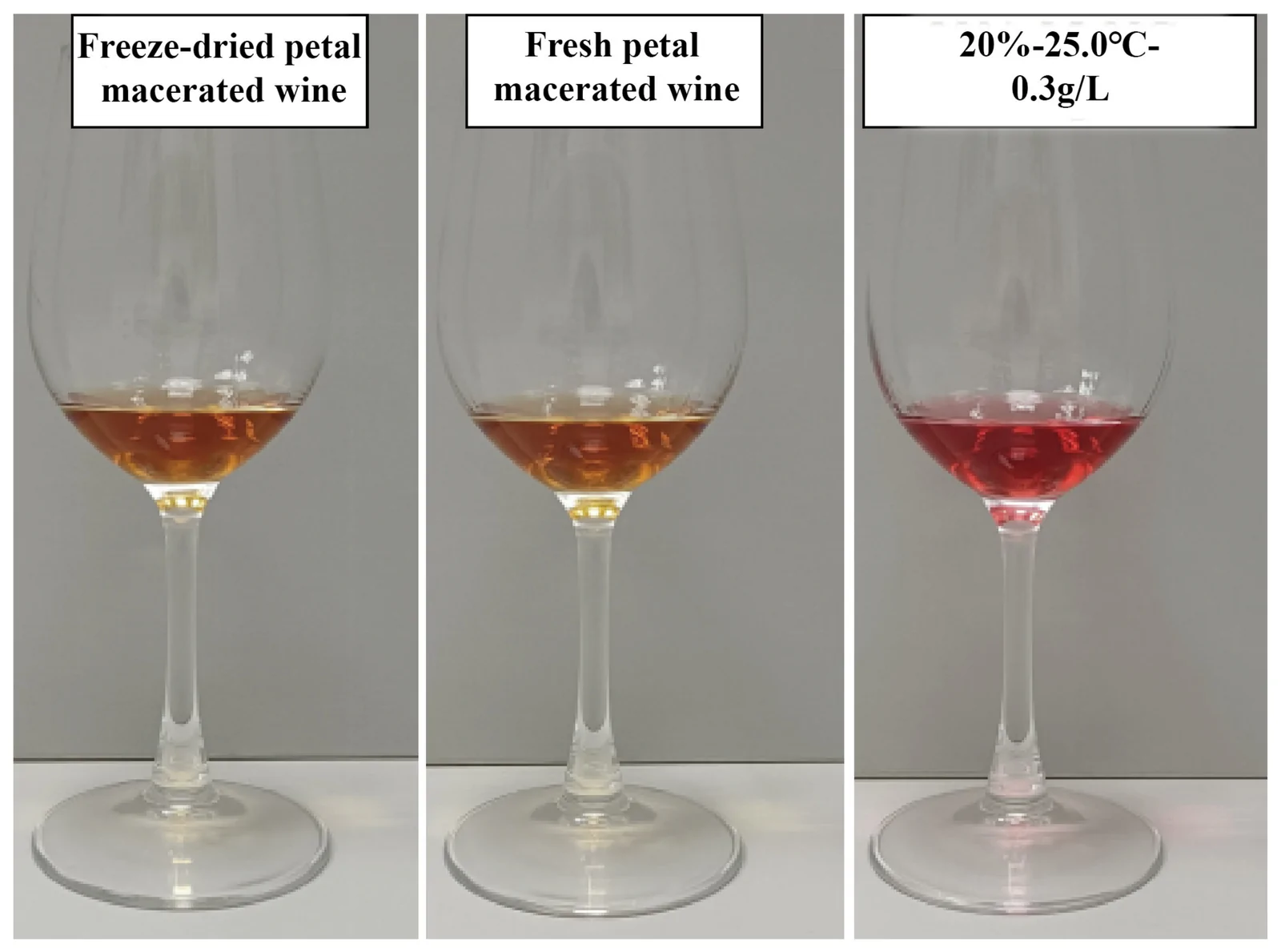
Visual appearance of three types of *Rosa rugosa* ‘Dianhong’ wines.

Based on the optimal maceration conditions for fresh *Rosa rugosa* ‘Dianhong’ petals (solid-to-solvent ratio 1:20, 40% *v*/*v* base alcohol, 60 days), freeze-dried petals were also subjected to maceration for comparison (Figure 8). As shown in Table 7, both fresh and freeze-dried petal-macerated wines contained higher soluble solids, total acids, and total flavonoids than honey-fermented wine, whereas the fermented wine had higher reducing sugar and total phenol levels. Significant differences in Δ*E* values were observed among the three wine types. The fermented wine exhibited a deep, bright, and transparent rose-red color with superior sensory quality. Furthermore, freeze-dried petal-macerated wine showed higher Δ*E* values, greater accumulation of total phenols, total flavonoids, and anthocyanins, and stronger DPPH radical scavenging capacity than fresh petal-macerated wine. These results indicate that freeze-drying effectively promoted the dissolution of antioxidant compounds from rose petals.

### 3.5. Analysis of Volatile Aroma Compounds in Rosa rugosa ‘Dianhong’ Petals and Three Kinds of Rose Wines

#### 3.5.1. Overall Volatile Profiles and TIC Spectra of Fresh Petals and Different Rose Wines

**Figure 9:**
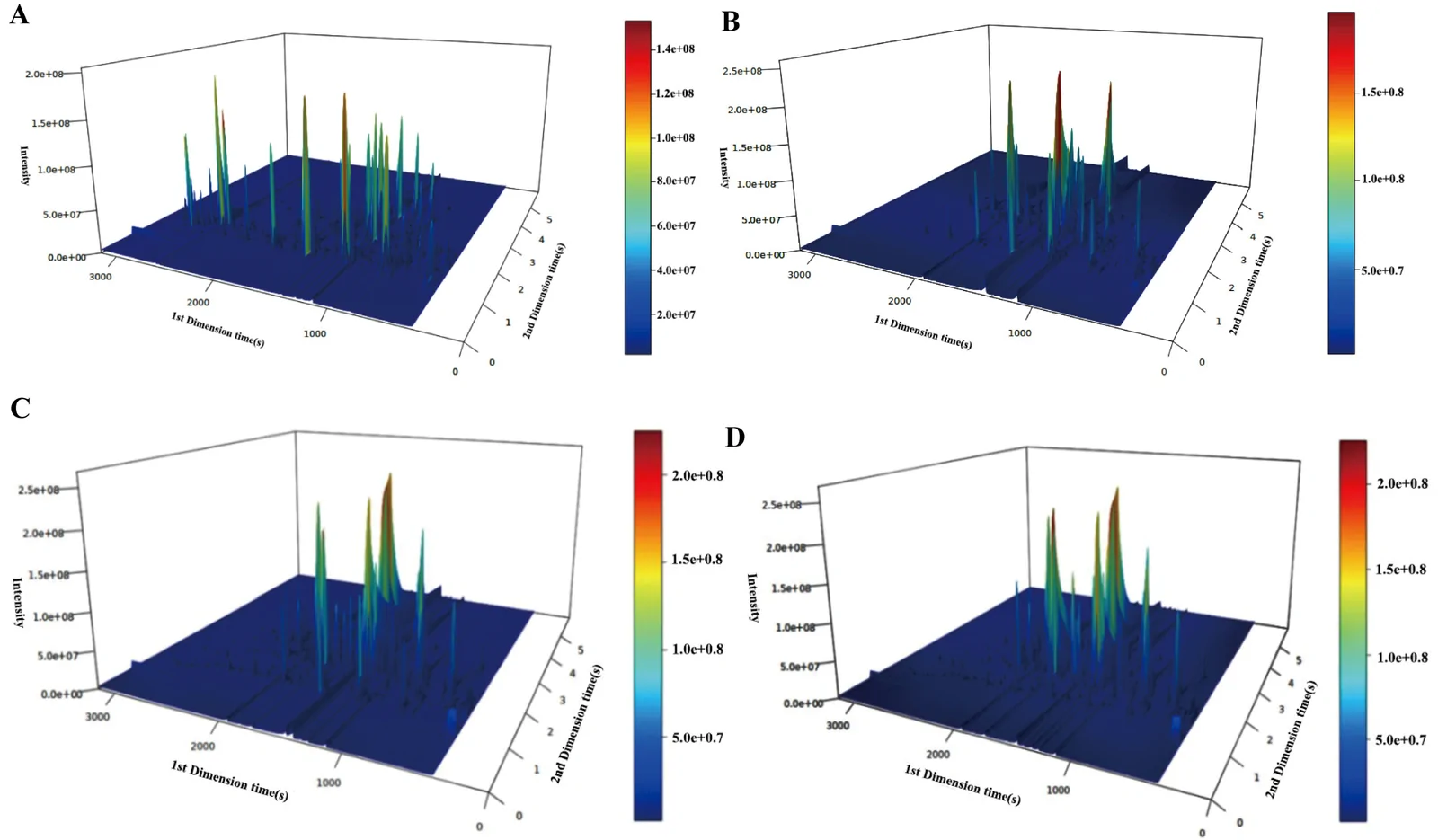
Total ion current (TIC) chromatograms of volatile aroma compounds and compositional analysis of *Rosa rugosa* ‘Dianhong’ samples. (**A**) Fresh Dianhong rose petals; (**B**) honey-fermented Dianhong rose wine; (**C**) fresh petal-macerated Dianhong rose wine; (**D**) freeze-dried petal-macerated Dianhong rose wine.

SPME-GC×GC-TOF-MS was used to analyze volatile aroma compounds in fresh *Rosa rugosa* ‘Dianhong’ petals, honey-fermented rose wine, fresh petal-macerated wine, and freeze-dried petal-macerated wine. Total ion current (TIC) chromatograms are shown in Figure 9, illustrating peak distribution, quantity, and signal intensity across samples. Characteristic aroma compounds were detected in all groups, confirming reliable identification of floral volatile components. The four samples differed markedly in peak number and signal intensity, indicating divergence in volatile composition between petals and wine products. A total of 136, 140, 195, and 186 volatile compounds accounting for 89.3%, 91.2%, 95.1%, and 94.7% of total peak area, respectively, were tentatively identified in fresh petals, fermented wine, fresh petal-macerated wine, and freeze-dried petal-macerated wine, respectively. The two macerated wines contained more volatile species than the fermented wine. Candidate key odorants, including benzaldehyde, phenethyl alcohol, citronellol, phenylacetaldehyde, rose oxide, and theaspirane, showed strong peaks and are candidate contributors to the typical rose scent. Freeze-dried petal-macerated wine exhibited higher peak intensities of phenethyl alcohol and citronellol than fresh petal-macerated wine, suggesting that maceration preserved and concentrated native floral volatiles.

#### 3.5.2. Diversity and Differential Distribution of Volatile Aroma Compounds in *Rosa rugosa* ‘Dianhong’ Petals and Rose Wine

**Figure 10:**
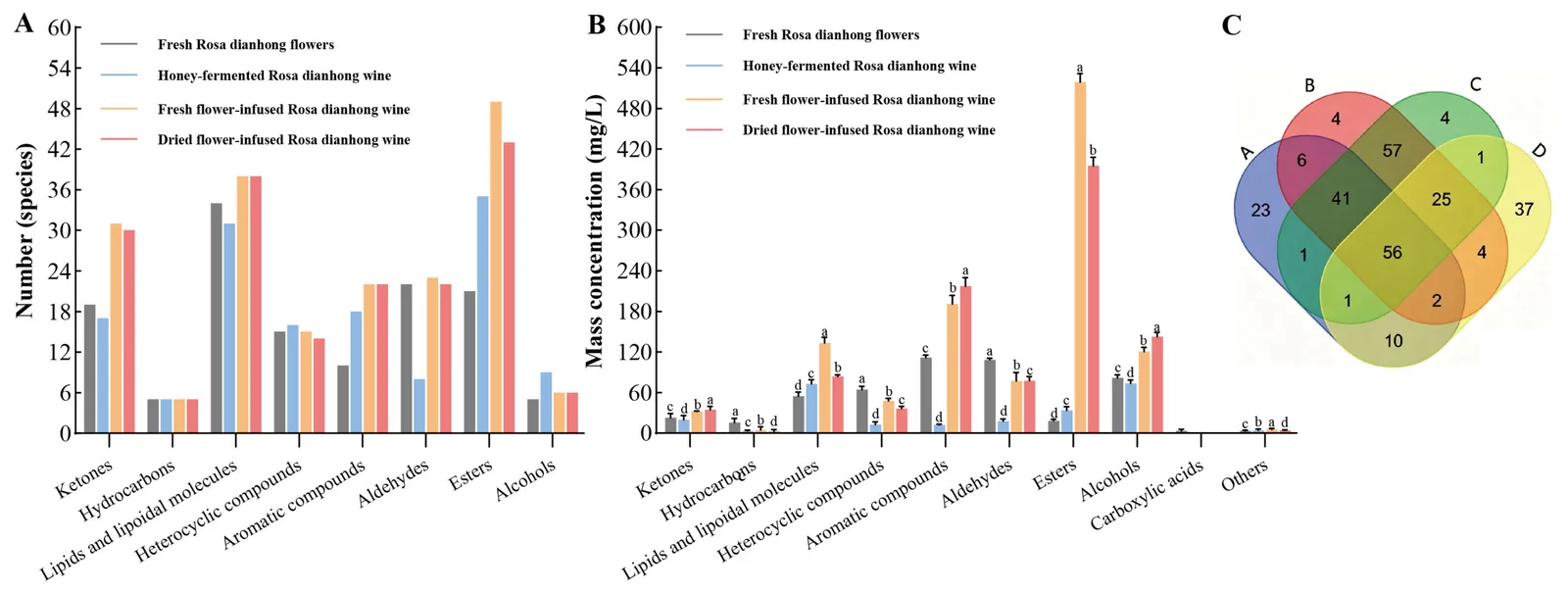
Determination of aroma components in fresh Dianhong rose and rose wine by SPME-GC×GC-TOF-MS. (**A**) Number of volatile compounds; (**B**) mass concentration of volatile compounds; (**C**) Venn diagram. Note: A = Dianhong rose honey-fermented wine; B = fresh Dianhong macerated rose wine; C = dried Dianhong macerated rose wine; D = fresh petals. Different lowercase letters indicate significant differences (*p* < 0.05).

To clarify the effects of winemaking processes on volatile substances in fresh *Rosa rugosa* ‘Dianhong’ petals, GC×GC-TOF-MS was applied to identify and quantify volatile components in fresh rose petals, honey-fermented wine, fresh petal-macerated wine, and freeze-dried petal-macerated wine. A total of 272 volatile compounds were tentatively detected across all samples and classified into 10 categories: 26 aromatic compounds, 56 lipids and lipid-like molecules, 60 esters, 13 alcohols, 46 ketones, 31 heterocyclic compounds, 27 aldehydes, 6 hydrocarbons, 2 carboxylic acids, and 5 other substances (Figure 10A). Venn diagram analysis (Figure 10C) revealed 56 volatile compounds common to all samples, constituting the common volatile basis of *Rosa rugosa* ‘Dianhong’ and its processed wine products; esters were the most abundant class contributors.

Significant differences in characteristic aroma substances were observed among wines prepared by different processing methods. Fermented wine contained higher citronellol levels than macerated wines, consistent with a better retention of the intrinsic floral fragrance of rose during fermentation. A total of 13 higher alcohols (>C2) were identified in rose wine, including phenethyl alcohol, 2-nonanol, 3-methyl-1-pentanol, 1-octen-3-ol, and benzyl alcohol; these compounds are associated with floral, green, rose-like, and citrus notes. As shown in Figure 10B, fresh rose petals contained higher concentrations of heterocyclic compounds than fermented and macerated wines, contributing floral, honey-like, and fruity flavors. A total of 27 aldehydes and 46 ketones were detected in all samples. Although these compounds exhibited relatively low contents and relatively low peak intensities, they imparted subtle honey, floral, caramel, fatty, and almond flavors to the wine matrix. Furfural was detected in both fresh petals and rose wine, with higher concentrations in macerated than in fermented wines.

Additionally, 26 aromatic compounds, such as benzaldehyde, ethyl phenylacetate, eugenol, and benzyl acetate, were identified in rose wine, playing a vital role in modulating the comprehensive aroma characteristics of fermented and macerated rose wine. These results demonstrate that different winemaking processes significantly affected the retention, transformation, and accumulation of volatile substances in *Rosa rugosa* ‘Dianhong’, thereby forming distinct aroma quality characteristics.

#### 3.5.3. OPLS-DA Analysis of Volatile Compounds in Fresh Petals and Wine of *Rosa rugosa* ‘Dianhong’

**Figure 11:**
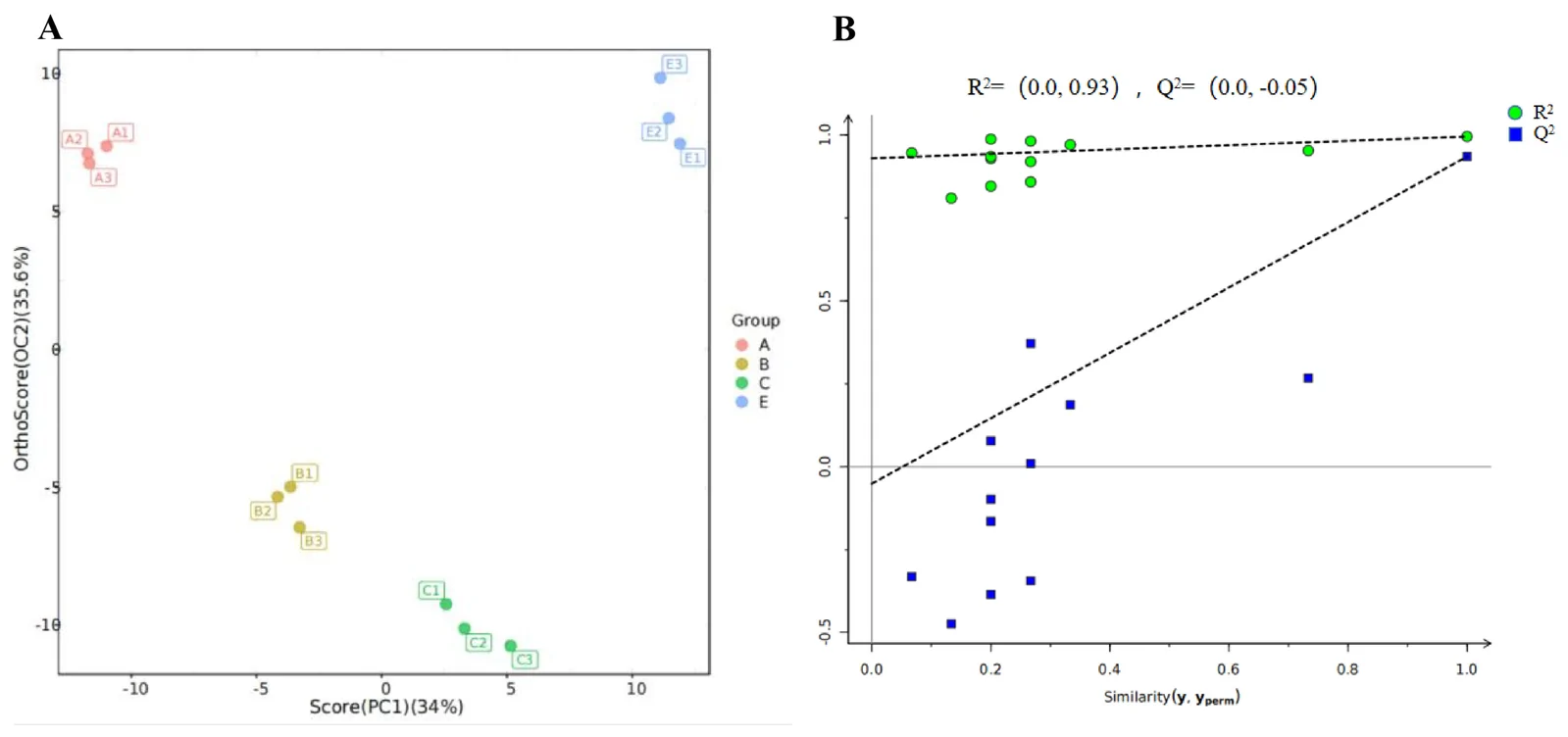
Orthogonal partial least squares discriminant analysis (OPLS-DA) and permutation test cross-validation of *Rosa rugosa* ‘Dianhong’ samples. (**A**) OPLS-DA score plot; (**B**) permutation test cross-validation plot.

OPLS-DA was performed on all volatile compounds to establish a model correlating compound content with sample groups (Figure 11A). This model effectively discriminated among fresh *Rosa rugosa* ‘Dianhong’ petals, honey-fermented wine, fresh petal-macerated wine, and freeze-dried petal-macerated wine. The model parameters were as follows: R^2^X = 0.756, R^2^Y = 0.995, and Q^2^ = 0.934. Both R^2^ and Q^2^ values exceeded 0.5, indicating acceptable model fit [32,33].

A permutation test was conducted for model validation (Figure 11B). The Q^2^ intercept on the vertical axis was below zero, confirming no overfitting and valid model performance. These results support the validity of the discrimination of the aroma profiles among fresh petals, fermented wine, and macerated wine products.

#### 3.5.4. Differential Analysis of Aroma Compounds in Fresh Petals and Wine of *Rosa rugosa* ‘Dianhong’

**Figure 12:**
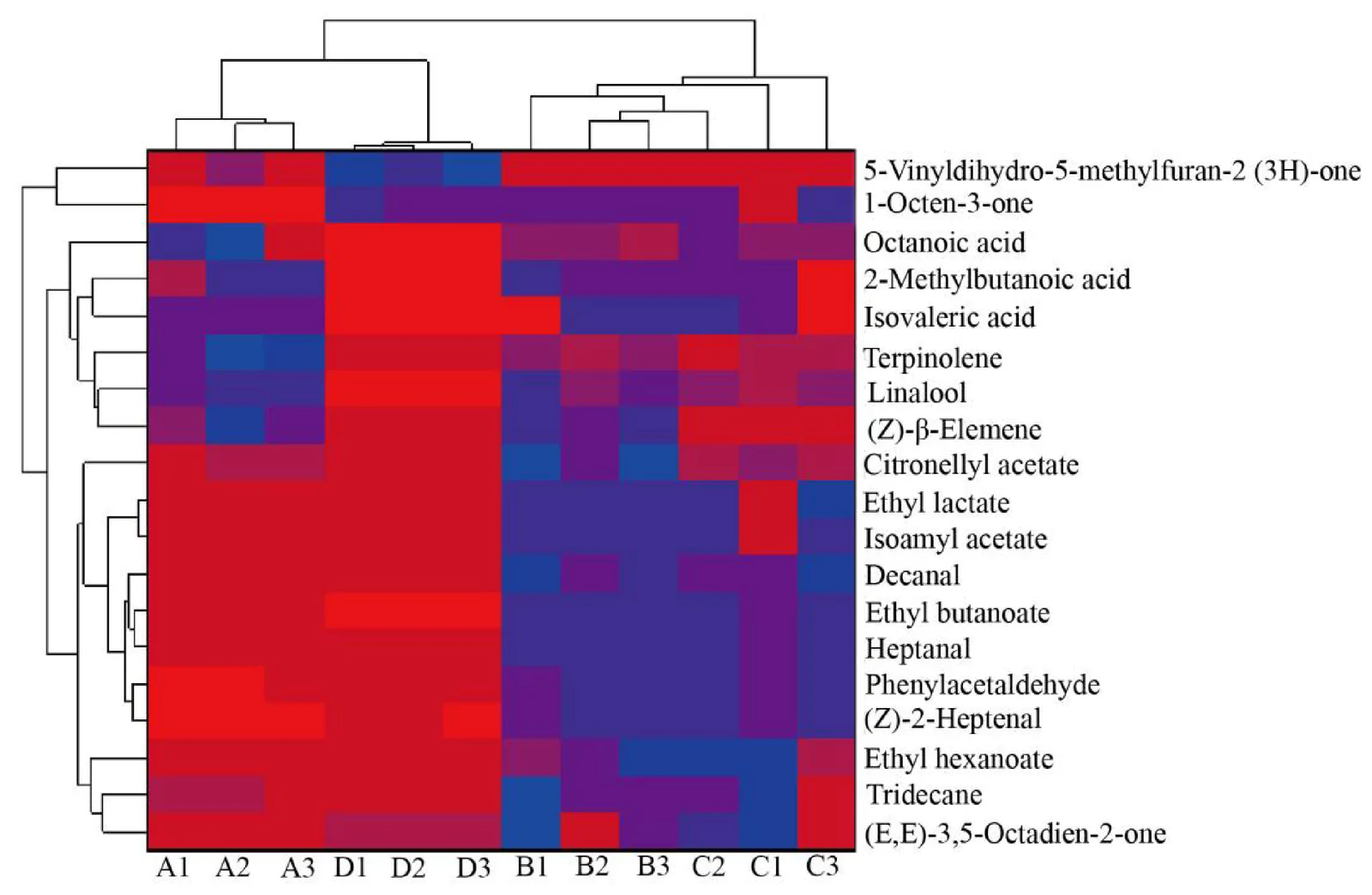
Clustered heatmap of differential aroma compounds from fresh Rosa Dianhong flowers and rose wine products. Note: A represents fresh *Rosa rugosa* ‘Dianhong’ petals; B represents fresh petal-macerated wine; C represents freeze-dried petal-macerated wine; D represents honey-fermented wine. The numbers 1, 2, and 3 denote three biological replicates of each sample group.

To further analyze the differences in individual aroma compounds between fresh *Rosa rugosa* ‘Dianhong’ petals and corresponding wine products, 19 differential volatile compounds were screened among the four sample groups using the criteria of *p* < 0.05 and VIP > 1, and a clustering heatmap was constructed (Figure 12). These differential metabolites consisted of seven lipids and lipid-like molecules, one heterocyclic compound, one hydrocarbon, four aldehydes, two ketones, and four esters.

Fresh petal-macerated wine and freeze-dried petal-macerated wine were clustered into the same group, indicating highly similar volatile profiles between the two macerated wine samples. Fresh petals and honey-fermented wine formed separate clusters. Five aroma compounds exhibited significantly higher contents in fresh petals, including linalool, ethyl butanoate, isovaleric acid, 2-methylbutanoic acid, and octanoic acid. By contrast, honey-fermented wine was rich in three characteristic volatiles: 1-octen-3-one, phenylacetaldehyde, and (Z)-2-heptenal. Most of these differential aroma substances contributed floral, fruity, fresh, and green sensory notes to the samples.

## 4. Discussion

This study connected three steps that are usually carried out separately: cultivar screening, process optimization, and aroma analysis. By following the selected cultivar from the petal to the bottled wine, this approach enabled the correlation of raw-material quality with processing conditions and the aroma chemistry of the final products. The discussion below follows the same sequence, with emphasis on how raw-material traits, processing conditions, and aroma chemistry determine the quality of the two types of rose wine, which differ fundamentally in how they are made.

The first stage examined whether petal biochemistry could predict winemaking suitability. *Rosa rugosa* ‘Dianhong’ and *Rosa centifolia* accumulated higher levels of fermentable sugars (Figure 2A)and, among the six cultivars evaluated, would require the least sugar supplementation to reach a target alcohol degree [34,35]. The weak acidity of all cultivars (pH 5.03–5.94, Figure 2C) required acid adjustment for optimal yeast performance [36]. The intense red coloration of *Rosa rugosa* ‘Dianhong’ (highest a* value, 72.46 ± 0.93) reflects its high anthocyanin accumulation, which is consistent with the significant positive correlation between the a* value and the total anthocyanin content [17], and copigmentation with flavonoids may further stabilize and intensify this coloration [37,38]. Conversely, *Rosa damascena* contained the lowest total flavonoid and total anthocyanin contents, consistent with its pale petals and low a* value. The flavonoid profile may be mechanistically linked to antioxidant capacity, because flavonoids scavenge radicals through hydroxyl-group donation and conjugated-electron stabilization; accordingly, total flavonoid content correlated significantly and positively with DPPH scavenging activity. The paradoxical inverse correlation between pH and flavonoid/anthocyanin contents, together with the positive correlation between total acids content and DPPH scavenging activity, suggests that the weakly acidic petal environment may enhance the stability, protonation state, and solubility of phenolic compounds [39,40,41]. Różyło et al. [42] reported 71.8 mg GAE/g total phenolics in *Rosa rugosa* petal powder, while Chen et al. [43] obtained 57.82 mg GAE/g for *Rosae rugosae* flos. These values agree with the high total phenolic, flavonoid, and anthocyanin contents of ‘Dianhong’ petals in the present study. All six cultivars showed DPPH scavenging rates above 50%, with *Rosa rugosa* ‘Dianhong’ the strongest (Figure 2F); among the six cultivars examined, it therefore combined the pigment, antioxidant, and sugar traits most relevant to beverage production [44,45].

The second stage investigated how the fermentation parameters affected the transfer of petal quality into the wine. The depletion of reducing sugars directly reflects yeast metabolic activity. An initial sugar concentration of 20% (*w*/*v*) yielded a wine with typical floral characteristics and the highest total sensory score (76.6 ± 0.8). Below this level, the osmotic support for alcohol formation is insufficient and the wine tends to be thin-bodied and atypical; above it, the elevated osmotic pressure restrains yeast activity, reduces the alcohol yield, and increases the residual sugar [46]. High-sugar musts also inhibit the leaching of phenolics and flavonoids from the petals. This phenomenon is consistent with previous reports that elevated sugar concentration increases must viscosity and osmotic pressure, which hampers mass transfer and weakens cell-membrane permeability, thereby retarding the release of intracellular phenolics [47]. The yeast inoculation dosage critically regulates the fermentation rate and flavor formation [48]. With increasing inoculum size, the contents of total phenols and total flavonoids first increased and then decreased, peaking at 0.30 g/L (sensory score 71.2 ± 1.7). At excessive cell densities (≥0.40 g/L), a larger fraction of the carbohydrates are diverted to biomass rather than ethanol, and the accumulation of by-products such as acetaldehyde and fusel alcohols can induce early yeast senescence and reduce antioxidant retention [49]. Temperature acts mainly through enzyme kinetics and yeast viability: 20 °C inhibited yeast growth and slowed the accumulation of alcohol and aroma compounds [50], whereas 30 °C accelerated sugar consumption and led to declines in phenolic and flavonoid contents. The 20–25 °C range effectively balanced fermentation efficiency with bioactive compound accumulation, with 25 °C giving the highest sensory score (76.1 ± 2.1). Orthogonal analysis ranked yeast inoculation dosage as the most influential factor (R = 0.280) and fermentation temperature as the least (R = 0.024), indicating that metabolic-flux distribution is more sensitive to cell density than to thermal energy within the tested range. Mechanistically, the superior aroma of the optimized fermented wine can be attributed to yeast metabolism: ethanol is produced via glycolysis, higher alcohols such as 2-phenylethanol are formed through the Ehrlich pathway, and acetate esters are synthesized from higher alcohols and GC–olfactometry by alcohol acetyltransferases; all of these pathways are sensitive to the temperature and inoculum size optimized above. The phenolic levels of the wines obtained in the present study are also comparable to those of other rose-derived wines. Cendrowski et al. [23] reported substantial polyphenol contents and antioxidant activity in wines produced from *Rosa rugosa* fruits, and Yang et al. [51] obtained a total phenolic content of 580.69 mg GAE/L in a pear-rose (*Rosa rugosa*) composite fermented wine, a magnitude comparable to the phenolic levels of the honey-fermented and macerated rose wines obtained here (Table 7).

For the macerated beverage, extraction rather than microbial metabolism governs quality formation. Increasing the solid-to-solvent ratio decreased wine lightness L* but increased redness a* and yellowness b*, reflecting the enhanced leaching of phenols and anthocyanins from rose petals [52]. Ethanol concentration and maceration duration jointly determined extraction efficiency: ethanol acts as a solvent that disrupts petal cell membranes and solubilizes polyphenols, which explains the peak of a* and total flavonoids at 40% *v*/*v* and their decline at higher concentrations, where excessive alcohol co-extracts interfering compounds and suppresses the perceived floral notes. Extended maceration (60 days) softened and disrupted petal cell walls, promoting continuous phenolic dissolution; beyond 60 days, however, enzymatic and chemical degradation of pigments became dominant. Endogenous polyphenol oxidase oxidizes o-diphenolic substrates to highly reactive o-quinones, which subsequently condense with anthocyanins to form brownish polymeric pigments [53]. Meanwhile, prolonged maceration promotes the condensation and polymerization of anthocyanins with other phenolics and acetaldehyde, destabilizing the red coloration and shifting the hue toward brown [54]; similarly, extended maceration has been reported to reduce the anthocyanin content of finished wines [55]. These coupled processes explain the decline in total phenols and flavonoids and the fading of wine color observed after 60 days, underscoring the need for precise temporal control in macerated wine production. The drying method also proved critical: freeze-drying preserves floral bioactive compounds and cell microstructure by sublimating ice at low temperature, thereby minimizing thermal degradation and oxidative damage, whereas air drying typically causes tissue shrinkage, browning, and volatile loss [56]. This explains why freeze-dried petals released more phenolics, flavonoids, and anthocyanins into the macerated wine than fresh petals (Table 7).

The two types of wine also acquired their aroma in different ways. Maceration extracted and retained a broad spectrum of native petal volatiles, with esters, lipids, and aromatic compounds forming the core of the aroma. Fermentation, in contrast, actively reshaped the aroma through yeast metabolism: the synthesis of esters and alcohols increased, aldehydes decreased, and some glycosidically bound volatiles were released, resulting in a more balanced aroma profile [57,58]. Fresh petal-macerated wine retained the largest number of volatile compounds and the most intense floral and fruity notes, whereas freeze-drying enhanced the accumulation of 2-phenylethanol and fresh petals better retained citronellol. The endogenous enzymes of intact fresh petals may catalyze favorable aroma transformations during maceration, and the acid-catalyzed conversion of terpenols during prolonged maceration may also play a role. Linalool, rose oxide, and eugenol were effectively retained during processing and together constitute candidate contributors to the characteristic aroma of Dianhong rose wines. This interpretation is consistent with the GC–olfactometry and odor activity value (OAV) data of Ong and Acree [59], which identified 2-phenylethanol, citronellol and phenethyl acetate as among the most odor-potent compounds in wines with a rose-like aroma, and with the floral volatile profiles of Rosa flowers reported by Quan et al. [40] and Wang et al. [60]. It should be noted, however, that SPME-GC×GC-TOF-MS measures the abundance rather than the sensory impact of volatiles. The odor activity value (OAV), the ratio of the concentration to the odor threshold, determines the actual contribution of a compound to the aroma [61,62]. The present results therefore identify candidate key odorants, and future work combining GC–olfactometry with OAV analysis is needed to confirm their sensory relevance.

Several limitations of this study should be acknowledged. First, all rose materials were sourced from a single experimental station during one growing season, so the effects of environmental variation on phytochemical profiles and wine quality remain uncharacterized, and the conclusions are strictly limited to the six cultivars and the processing conditions evaluated. Second, volatile compound identification relied on mass-spectral library matching and retention-index verification without confirmation by authentic standards, and odor activity values were not determined, so the sensory contribution of individual compounds remains inferential. Third, the stability of bioactive compounds, color parameters, and volatile profiles during long-term storage was not investigated. Fourth, sensory evaluation was performed by a modestly sized trained panel, and quantitative descriptive analysis with a larger panel would further strengthen the sensory conclusions. Finally, all optimizations were conducted at laboratory scale; industrial translation will require pilot-scale validation of the optimized fermentation and maceration protocols, assessment of batch consistency and scalability, economic feasibility analysis, product stability testing, and compliance with relevant food regulations. Addressing these gaps through multi-site, multi-year trials, storage kinetics, GC–O/OAV analysis, and scale-up studies will support the transition from laboratory scale optimization to industrial practice.

## 5. Conclusions

Among the six cultivars evaluated under the present experimental conditions, *Rosa rugosa* ‘Dianhong’ exhibited the highest contents of phenolics, flavonoids, and anthocyanins and the strongest antioxidant capacity, and was therefore selected for beverage development. For the honey-fermented wine, 20% (*w*/*v*) initial sugar, 0.30 g/L yeast inoculum, and a 25 °C fermentation temperature yielded wine with 10.1% (*v*/*v*) alcohol and the highest sensory score among the treatments tested; for the macerated beverage, a solid-to-solvent ratio of 1:20 (*w*/*v*), 40% (*v*/*v*) base alcohol, and a 60-day maceration were selected as the best conditions within the tested single-factor ranges. A total of 272 volatile compounds were tentatively identified, with esters and alcohols as the most abundant volatile classes, but fermentation promoted their formation whereas maceration better retained native petal volatiles. Integrating cultivar screening, process optimization, and aroma profiling provides a laboratory-scale framework for developing rose-based beverages with defined quality targets; pilot-scale validation, storage-stability assessment, and odor-activity analysis are warranted prior to industrial application.

## Figures and Tables

**Table 1:** Detection index results of ‘Dianhong’ rose honey wine with different initial sugar contents, yeast inoculation amounts and fermentation temperatures (aged for 1 month).

Initial Sugar Concentration (%)	L*	a*	b*	Total Phenols (mg/g)	Total Flavonoids (mg/g)	Soluble Solids (%)
16	70.18 ± 1.36 ^e^	41.23 ± 3.12 ^a^	16.33 ± 1.73 ^e^	9.41 ± 0.52 ^d^	5.23 ± 2.30 ^c^	6.15 ± 1.10 ^d^
18	72.08 ± 1.77 ^d^	36.36 ± 1.63 ^b^	17.94 ± 2.21 ^d^	9.49 ± 1.28 ^c^	4.39 ± 1.07 ^e^	6.88 ± 1.04 ^c^
20	72.77 ± 2.00 ^c^	34.75 ± 1.28 ^c^	18.03 ± 0.92 ^c^	14.59 ± 1.15 ^a^	6.11 ± 1.24 ^a^	6.88 ± 1.05 ^c^
22	74.03 ± 1.13 ^b^	31.52 ± 1.08 ^d^	20.27 ± 2.01 ^b^	14.59 ± 1.48 ^a^	6.03 ± 1.50 ^b^	8.00 ± 1.11 ^b^
24	75.54 ± 1.21 ^a^	27.31 ± 1.11 ^e^	22.31 ± 1.43 ^a^	10.83 ± 0.94 ^b^	4.66 ± 1.70 ^d^	9.55 ± 1.08 ^a^
Yeast inoculation dosage (g/L)	L*	a*	b*	Total phenols (mg/g)	Total flavonoids (mg/g)	Soluble solids (%)
0.10	79.00 ± 1.39 ^e^	10.86 ± 1.07 ^d^	34.97 ± 2.57 ^d^	10.78 ± 1.40 ^d^	3.20 ± 2.49 ^e^	8.27 ± 1.19 ^b^
0.20	80.20 ± 1.53 ^d^	12.00 ± 1.70 ^b^	36.05 ± 1.69 ^b^	11.02 ± 1.47 ^c^	3.86 ± 1.19 ^c^	8.22 ± 1.04 ^c^
0.30	80.59 ± 2.18 ^c^	15.21 ± 3.80 ^a^	36.46 ± 0.92 ^a^	11.64 ± 1.18 ^a^	4.06 ± 1.06 ^a^	8.40 ± 1.06 ^a^
0.40	81.06 ± 1.02 ^b^	11.30 ± 1.03 ^c^	35.16 ± 2.03 ^c^	11.34 ± 2.56 ^b^	4.03 ± 1.27 ^b^	7.92 ± 2.04 ^d^
0.50	82.23 ± 1.53 ^a^	9.31 ± 1.41 ^e^	33.97 ± 0.88 ^e^	10.28 ± 1.52 ^e^	3.63 ± 2.09 ^d^	7.93 ± 1.01 ^d^
Fermentation temperature (°C)	L*	a*	b*	Total phenols (mg/g)	Total flavonoids (mg/g)	Soluble solids (%)
20	81.49 ± 1.27 ^a^	7.74 ± 2.68 ^e^	40.06 ± 2.02 ^a^	11.27 ± 1.37 ^e^	4.09 ± 1.09 ^d^	7.95 ± 1.05 ^d^
22.5	80.44 ± 1.05 ^b^	10.43 ± 1.43 ^d^	38.33 ± 2.64 ^b^	11.68 ± 1.14 ^d^	4.16 ± 1.17 ^c^	7.77 ± 1.04 ^e^
25	73.44 ± 2.03 ^e^	30.91 ± 1.23 ^a^	22.81 ± 1.23 ^e^	13.81 ± 1.24 ^b^	6.23 ± 2.17 ^a^	8.04 ± 1.05 ^c^
27.5	73.75 ± 1.04 ^d^	29.02 ± 2.36 ^b^	24.78 ± 0.97 ^d^	13.83 ± 2.64 ^a^	6.23 ± 1.30 ^a^	8.15 ± 2.16 ^b^
30	75.69 ± 2.02 ^c^	24.05 ± 1.00 ^c^	28.53 ± 1.02 ^c^	13.51 ± 1.41 ^c^	5.69 ± 2.06 ^b^	8.25 ± 1.27 ^a^

Note: Different lowercase letters in the same column of data indicate significant differences (*p* < 0.05).

**Table 2:** Sensory evaluation scores of *Rosa rugosa* ‘Dianhong’ fermented honey wine under different fermentation conditions.

Initial Sugar Concentration (%)	Color	Clarity	Rose Floral Aroma	Harmony of Floral and Wine Aroma	Palate Fullness & Balance	Honey Flavor	Rose Aroma Flavor	Typical Rose Wine Character	Total Score
16	8.5 ± 0.3 ^a^	8.6 ± 0.2 ^a^	15.2 ± 0.3 ^a^	7.7 ± 0.6 ^b^	14.8 ± 0.1 ^c^	5.6 ± 0.5 ^d^	7.7 ± 0.7 ^a^	7.1 ± 0.4 ^c^	75.2 ± 0.9 ^b^
18	7.6 ± 0.4 ^b^	8.2 ± 0.9 ^b^	14.7 ± 0.2 ^b^	8.2 ± 0.2 ^a^	14.8 ± 0.1 ^c^	6.2 ± 0.3 ^c^	7.4 ± 0.1 ^b^	7.4 ± 0.2 ^b^	73.9 ± 1.5 ^c^
20	7.4 ± 1.3 ^c^	8.2 ± 0.4 ^b^	14.2 ± 0.2 ^c^	7.2 ± 0.3 ^c^	17.4 ± 0.4 ^a^	6.9 ± 1.0 ^a^	7.5 ± 0.1 ^b^	7.8 ± 0.5 ^a^	76.6 ± 0.8 ^a^
22	5.7 ± 2.2 ^d^	7.5 ± 0.5 ^c^	11.8 ± 0.8 ^e^	6.4 ± 0.2 ^d^	14.9 ± 0.2 ^bc^	6.1 ± 0.2 ^c^	6.3 ± 0.4 ^d^	6.8 ± 0.2 ^d^	65.5 ± 2.4 ^d^
24	4.4 ± 0.5 ^e^	6.5 ± 0.4 ^d^	12.5 ± 0.5 ^d^	6.1 ± 0.2 ^e^	15.0 ± 0.2 ^b^	6.6 ± 0.8 ^b^	6.7 ± 0.2 ^c^	6.6 ± 0.5 ^e^	64.5 ± 1.3 ^e^
Yeast inoculation dosage (g/L)	Color	Clarity	Rose floral aroma	Harmony of floral and wine aroma	Palate fullness & balance	Honey flavor	Rose aroma flavor	Typical rose wine character	Total score
0.1	6.2 ± 0.8 ^c^	7.4 ± 0.4 ^c^	12.5 ± 0.4 ^d^	7.2 ± 0.2 ^b^	14.9 ± 0.2 ^c^	5.8 ± 0.4 ^d^	6.1 ± 0.5 ^c^	6.8 ± 0.3 ^ab^	66.9 ± 1.1 ^b^
0.2	7.5 ± 1.2 ^a^	8.4 ± 0.9 ^a^	16.5 ± 0.3 ^a^	8.3 ± 0.2 ^a^	16.2 ± 0.5 ^a^	6.9 ± 0.7 ^a^	6.8 ± 0.1 ^b^	6.7 ± 0.4 ^c^	65.6 ± 1.0 ^c^
0.3	7.2 ± 1.5 ^b^	7.9 ± 0.6 ^b^	14.0 ± 0.1 ^b^	7.2 ± 0.3 ^b^	15.9 ± 0.2 ^b^	6.4 ± 0.4 ^b^	7.0 ± 0.4 ^a^	6.9 ± 0.3 ^a^	71.2 ± 1.7 ^a^
0.4	5.7 ± 2.2 ^d^	7.5 ± 0.5 ^c^	11.8 ± 1.8 ^e^	6.4 ± 0.2 ^c^	14.9 ± 0.2 ^c^	6.1 ± 0.2 ^c^	7.0 ± 0.7 ^a^	6.8 ± 0.5 ^ab^	65.7 ± 2.3 ^c^
0.5	4.3 ± 0.2 ^e^	7.4 ± 0.6 ^c^	13.0 ± 0.1 ^c^	6.1 ± 0.9 ^d^	14.7 ± 0.1 ^d^	6.5 ± 0.1 ^b^	6.1 ± 0.1 ^c^	6.5 ± 0.1 ^d^	64.6 ± 2.0 ^d^
Fermentation temperature (°C)	Color	Clarity	Rose floral aroma	Harmony of floral and wine aroma	Palate fullness & balance	Honey flavor	Rose aroma flavor	Typical rose wine character	Total score
20	3.7 ± 0.2 ^d^	7.2 ± 0.1 ^c^	12.1 ± 1.3 ^d^	7.1 ± 0.6 ^d^	14.5 ± 0.7 ^d^	6.3 ± 0.2 ^c^	6.2 ± 0.4 ^c^	6.8 ± 0.2 ^d^	63.7 ± 3.7 ^d^
22.5	3.3 ± 0.4 ^e^	6.0 ± 0.9 ^e^	10.8 ± 0.8 ^e^	7.4 ± 0.4 ^c^	13.9 ± 0.2 ^e^	5.8 ± 0.1 ^d^	5.7 ± 0.2 ^d^	6.3 ± 0.2 ^e^	59.1 ± 1.1 ^e^
25	7.7 ± 0.4 ^a^	8.6 ± 0.2 ^a^	14.6 ± 1.0 ^a^	7.7 ± 0.2 ^b^	16.5 ± 0.6 ^a^	6.5 ± 0.1 ^b^	6.9 ± 0.1 ^a^	7.6 ± 0.4 ^b^	76.1 ± 2.1 ^a^
27.5	6.9 ± 0.6 ^b^	7.9 ± 0.2 ^b^	12.7 ± 0.9 ^c^	7.0 ± 0.5 ^d^	15.4 ± 0.5 ^c^	5.6 ± 0.8 ^e^	6.7 ± 0.1 ^b^	7.3 ± 0.5 ^c^	69.4 ± 1.1 ^c^
30	5.2 ± 1.5 ^c^	7.0 ± 0.6 ^d^	13.2 ± 0.3 ^b^	8.0 ± 0.2 ^a^	15.6 ± 0.8 ^b^	7.2 ± 0.1 ^a^	7.0 ± 0.1 ^a^	7.9 ± 0.1 ^a^	71.0 ± 2.7 ^b^

Note: All values are expressed as mean ± standard deviation. Different lowercase superscript letters in the same column denote significant differences among treatments at (*p* < 0.05).

**Table 3:** Determination indices of *Rosa rugosa* ‘Dianhong’ fermented honey wine under different fermentation processes.

Fermentation Process	Total Phenols (mg/g)	Total Flavonoids (mg/g)	Soluble Solids (%)	Δ*E*	Sensory Score
18%-22.5 °C-0.20 g/L	11.76 ± 0.73 ^h^	6.26 ± 0.21 ^h^	6.73 ± 0.05 ^f^	60.00 ± 2.51 ^f^	73.20 ± 1.13 ^h^
20%-22.5 °C-0.20 g/L	12.36 ± 0.56 ^g^	6.38 ± 0.41 ^g^	7.38 ± 0.05 ^d^	56.98 ± 1.18 ^h^	73.90 ± 2.40 ^e^
22%-22.5 °C-0.20 g/L	13.69 ± 0.77 ^b^	7.29 ± 1.70 ^b^	8.35 ± 0.13 ^a^	54.04 ± 1.98 ^i^	75.20 ± 1.13 ^c^
18%-25.0 °C-0.30 g/L	13.19 ± 0.61 ^f^	7.12 ± 0.20 ^d^	6.73 ± 0.10 ^f^	67.87 ± 1.38 ^b^	73.60 ± 0.42 ^g^
20%-25.0 °C-0.30 g/L	14.39 ± 0.25 ^a^	7.35 ± 0.36 ^a^	7.53 ± 0.05 ^c^	68.36 ± 0.40 ^a^	77.65 ± 0.21 ^a^
22%-25.0 °C-0.30 g/L	13.27 ± 0.60 ^e^	6.98 ± 0.17 ^e^	8.35 ± 0.06 ^a^	60.62 ± 0.17 ^e^	73.80 ± 2.97 ^f^
18%-27.5 °C-0.40 g/L	13.58 ± 0.20 ^c^	7.23 ± 0.78 ^c^	6.83 ± 0.05 ^e^	62.24 ± 0.77 ^d^	73.90 ± 1.84 ^e^
20%-27.5 °C-0.40 g/L	13.42 ± 0.55 ^d^	6.81 ± 0.25 ^f^	7.53 ± 0.05 ^c^	62.45 ± 0.69 ^c^	75.00 ± 3.82 ^d^
22%-27.5 °C-0.40 g/L	13.70 ± 0.62 ^b^	6.98 ± 0.11 ^e^	8.25 ± 0.06 ^b^	59.62 ± 0.56 ^g^	75.30 ± 3.11 ^b^

Note: Different lowercase superscript letters within the same column indicate significant differences among samples at (*p* < 0.05).

**Table 4:** L_9_(3^4^) Orthogonal test results of *Rosa rugosa* ‘Dianhong’ fermented honey wine.

Test No	Factor A	Factor B	Factor C	Total Score
1	1	1	1	4.30
2	1	2	2	4.63
3	1	3	3	4.61
4	2	1	1	4.40
5	2	2	2	4.90
6	2	3	3	4.64
7	3	1	1	4.70
8	3	2	2	4.71
9	3	3	3	4.73
K1	4.587	4.467	4.643	——
K2	4.717	4.747	4.667	——
K3	4.570	4.660	4.657	——
Range R	0.147	0.280	0.024	

Note: The comprehensive score was calculated by the summation of the min–max-normalized scores (0–1) of the quality indices (physicochemical indices, color, antioxidant capacity and the sensory total score on a 5-point scale; see Section 2.4). Factor A, initial sugar concentration; Factor B, yeast inoculation dosage; Factor C, fermentation temperature. K1, K2 and K3 are the mean comprehensive scores at each level; R is the range.

**Table 5:** Detection index results of *Rosa rugosa* ‘Dianhong’ macerated wine under varied solid-to-solvent ratios, base wine alcohol concentrations and maceration periods.

Solid-to-Solvent Ratio	L*	a*	b*	Total Phenols (mg/g)	Total Flavonoids (mg/g)	Soluble Solids (%)
1:10	59.89 ± 0.49^5^	25.36 ± 0.77 ^a^	82.12 ± 0.98 ^a^	17.95 ± 1.12 ^a^	15.65 ± 0.58 ^a^	17.00 ± 0.17 ^c^
1:20	76.05 ± 0.38 ^d^	8.65 ± 0.27 ^b^	54.42 ± 1.10 ^b^	10.24 ± 0.54 ^b^	8.04 ± 0.20 ^b^	17.17 ± 0.12 ^bc^
1:30	83.72 ± 0.29 ^c^	2.20 ± 0.19 ^c^	36.06 ± 0.55 ^c^	7.86 ± 0.73 ^c^	5.55 ± 0.68 ^c^	17.22 ± 0.04 ^a^
1:40	86.64 ± 0.46 ^b^	0.95 ± 0.24 ^d^	26.20 ± 1.05 ^d^	5.85 ± 0.13 ^d^	4.12 ± 0.25 ^d^	17.18 ± 0.04 ^b^
1:50	88.55 ± 0.02 ^a^	0.41 ± 0.17 ^e^	19.83 ± 0.30 ^e^	4.96 ± 0.26 ^e^	3.54 ± 0.17 ^e^	17.17 ± 0.08 ^bc^
Alcohol Concentration (%*v*/*v*)	L*	a*	b*	Total phenols (mg/g)	Total flavonoids (mg/g)	Soluble solids (%)
35	83.46 ± 0.02 ^e^	3.21 ± 0.08 ^b^	37.30 ± 0.17 ^a^	8.31 ± 0.19 ^d^	4.34 ± 0.17 ^b^	12.40 ± 0.09 ^e^
40	83.54 ± 0.10 ^d^	3.38 ± 0.15 ^a^	36.09 ± 0.11 ^b^	8.54 ± 0.43 ^b^	4.40 ± 0.06 ^a^	13.63 ± 0.19 ^d^
45	84.24 ± 0.29 ^c^	2.94 ± 0.03 ^c^	33.08 ± 1.36 ^c^	8.26 ± 0.19 ^e^	4.23 ± 0.24 ^c^	14.68 ± 0.26 ^c^
50	85.15 ± 0.06 ^b^	2.49 ± 0.08 ^d^	29.17 ± 0.05 ^d^	8.51 ± 0.18 ^c^	4.09 ± 0.25 ^e^	15.85 ± 0.05 ^b^
55	85.83 ± 0.49 ^a^	2.28 ± 0.23 ^e^	25.69 ± 1.56 ^e^	8.65 ± 0.38 ^a^	4.14 ± 0.34 ^d^	16.87 ± 0.12 ^a^
Maceration Period (d)	L*	a*	b*	Total phenols (mg/g)	Total flavonoids (mg/g)	Soluble solids (%)
20	85.07 ± 0.40 ^b^	1.68 ± 0.53 ^e^	22.88 ± 4.08 ^e^	6.99 ± 0.21 ^e^	3.57 ± 0.44 ^c^	16.35 ± 0.15 ^e^
40	86.63 ± 1.37 ^a^	2.24 ± 0.22 ^d^	22.92 ± 0.95 ^d^	7.81 ± 0.59 ^d^	4.12 ± 0.46 ^b^	16.88 ± 0.16 ^d^
60	83.39 ± 0.27 ^c^	3.02 ± 1.15 ^b^	34.08 ± 1.36 ^c^	9.02 ± 0.21 ^a^	5.12 ± 0.30 ^a^	17.35 ± 0.10 ^a^
80	83.04 ± 0.04 ^d^	2.97 ± 0.03 ^c^	34.15 ± 0.21 ^b^	8.76 ± 0.16 ^c^	3.43 ± 0.11 ^d^	17.18 ± 0.18 ^c^
100	82.88 ± 0.19 ^e^	3.09 ± 0.06 ^a^	34.28 ± 0.59 ^a^	8.83 ± 0.10 ^b^	3.40 ± 0.16 ^e^	17.15 ± 0.28 ^b^

Note: Different labeled letters in the same column of data indicate significant differences (*p* < 0.05).

**Table 6:** Sensory scores of *Rosa rugosa* ‘Dianhong’ macerated wine under varied solid-to-solvent ratios, base wine alcohol concentrations and maceration periods.

Solid-to-Solvent Ratio	Color	Clarity	Rose Floral Aroma	Harmony of Floral and Wine Aroma	Palate Fullness & Balance	Sweet Flavor	Rose Aroma Flavor	Typical Rose Wine Character	Total Score
1:10	7.1 ± 1.5 ^a^	5.4 ± 0.9 ^e^	12.9 ± 0.7 ^b^	7.4 ± 0.4 ^b^	14.1 ± 0.7 ^c^	6.5 ± 0.1 ^b^	7.5 ± 0.3 ^a^	7.3 ± 0.1 ^a^	68.4 ± 0.9 ^b^
1:20	6.8 ± 0.2 ^b^	6.0 ± 0.2 ^d^	13.2 ± 1.3 ^a^	7.7 ± 0.1 ^a^	15.3 ± 0.6 ^a^	6.6 ± 0.2 ^a^	7.2 ± 0.2 ^b^	6.9 ± 0.2 ^b^	69.4 ± 1.6 ^a^
1:30	4.9 ± 1.2 ^c^	7.2 ± 1.2 ^b^	11.4 ± 0.3 ^c^	5.6 ± 0.6 ^c^	14.0 ± 0.5 ^d^	6.0 ± 0.1 ^c^	5.7 ± 0.5 ^c^	6.2 ± 0.1 ^c^	60.8 ± 2.3 ^c^
1:40	3.5 ± 1.3 ^d^	6.9 ± 1.2 ^c^	10.6 ± 0.4 ^d^	5.5 ± 0.1 ^d^	13.2 ± 1.3 ^e^	5.4 ± 1.5 ^e^	5.4 ± 0.6 ^c^	5.4 ± 0.6 ^e^	55.7 ± 3.8 ^e^
1:50	3.1 ± 0.4 ^e^	7.5 ± 0.6 ^a^	10.7 ± 0.8 ^e^	5.6 ± 0.4 ^c^	14.5 ± 0.2 ^b^	5.9 ± 0.6 ^d^	5.3 ± 0.8 ^c^	5.8 ± 0.2 ^d^	58.2 ± 0.8 ^d^
Alcohol Concentration (%*v*/*v*)	Color	Clarity	Rose floral aroma	Harmony of floral and wine aroma	Palate fullness & balance	sweet flavor	Rose aroma flavor	Typical rose wine character	Total score
35	4.0 ± 0.1 ^b^	7.0 ± 0.4 ^e^	12.9 ± 0.4 ^c^	6.8 ± 0.3 ^e^	14.7 ± 1.3 ^d^	6.6 ± 0.4 ^d^	6.4 ± 0.5 ^b^	6.3 ± 0.1 ^c^	64.7 ± 1.6 ^d^
40	4.8 ± 0.6 ^a^	7.6 ± 0.3 ^b^	14.6 ± 0.5 ^a^	7.5 ± 0.4 ^b^	15.4 ± 0.4 ^b^	7.3 ± 0.5 ^a^	6.6 ± 0.6 ^a^	6.7 ± 0.4 ^a^	70.5 ± 2.1 ^a^
45	4.0 ± 0.4 ^b^	7.8 ± 0.5 ^a^	13.7 ± 0.4 ^b^	7.3 ± 0.4 ^c^	15.5 ± 0.3 ^a^	7.1 ± 0.3 ^b^	6.6 ± 0.1 ^a^	6.5 ± 0.6 ^b^	68.3 ± 0.5 ^b^
50	3.1 ± 0.5 ^c^	7.5 ± 0.1 ^c^	13.7 ± 0.1 ^b^	7.9 ± 0.9 ^a^	14.1 ± 0.7 ^e^	6.9 ± 0.8 ^c^	6.6 ± 0.1 ^a^	6.2 ± 0.2 ^d^	65.7 ± 1.3 ^c^
55	3.1 ± 0.6 ^c^	7.4 ± 0.0 ^d^	12.6 ± 0.3 ^d^	7.1 ± 0.6 ^d^	14.8 ± 1.0 ^c^	6.5 ± 0.4 ^e^	6.3 ± 0.7 ^c^	6.7 ± 0.4 ^a^	64.3 ± 2.0 ^e^
Maceration Period (d)	Color	Clarity	Rose floral aroma	Harmony of floral and wine aroma	Palate fullness & balance	sweet flavor	Rose aroma flavor	Typical rose wine character	Total score
20	3.6 ± 0.9 ^d^	7.8 ± 0.0 ^b^	13.1 ± 0.8 ^b^	6.6 ± 0.2 ^c^	16.2 ± 1.0 ^b^	7.0 ± 0.2 ^c^	6.5 ± 0.4 ^b^	6.0 ± 0.1 ^d^	66.7 ± 0.9 ^c^
40	3.8 ± 0.4 ^c^	7.8 ± 0.1 ^b^	12.6 ± 0.0 ^c^	6.6 ± 0.1 ^c^	15.5 ± 0.8 ^c^	6.3 ± 0.7 ^d^	5.9 ± 0.2 ^d^	6.3 ± 0.2 ^b^	64.0 ± 1.1 ^d^
60	5.3 ± 0.1 ^a^	7.6 ± 0.0 ^c^	13.2 ± 0.4 ^a^	6.8 ± 0.1 ^b^	16.4 ± 1.5 ^a^	7.3 ± 0.4 ^a^	6.8 ± 0.1 ^a^	7.1 ± 0.4 ^a^	70.3 ± 1.1 ^a^
80	3.3 ± 0.2 ^e^	7.5 ± 0.6 ^d^	11.3 ± 0.2 ^d^	6.1 ± 0.4 ^d^	14.9 ± 0.9 ^d^	6.0 ± 0.3 ^e^	5.5 ± 0.1 ^e^	6.0 ± 0.1 ^d^	60.4 ± 2.0 ^e^
100	4.7 ± 0.4 ^b^	8.1 ± 0.2 ^a^	13.1 ± 0.2 ^b^	7.9 ± 0.4 ^a^	14.7 ± 1.2 ^e^	7.2 ± 0.3 ^b^	6.0 ± 0.4 ^c^	6.2 ± 0.4 ^c^	67.7 ± 2.3 ^b^

Note: All values are expressed as mean ± standard deviation. Different lowercase superscript letters in the same column denote significant differences among treatments at (*p* < 0.05).

**Table 7:** Physicochemical and antioxidant indicators of *Rosa rugosa* ‘Dianhong’ wines.

Wine Sample	Reducing Sugars (g/L)	Soluble Solids (%)	Total Acids (g/L)	Δ*E*	Total Phenols (mg/g)	Total Flavonoids (mg/g)	Total Anthocyanins (mg/g)
Fresh petal fermented wine	9.87 ± 0.58 ^a^	7.53 ± 0.05 ^c^	3.40 ± 0.02 ^c^	62.24 ± 0.77 ^a^	14.39 ± 0.25 ^a^	7.12 ± 0.20 ^c^	6.83 ± 0.42 ^a^
Fresh petal-macerated wine	5.69 ± 0.77 ^b^	14.68 ± 0.15 ^b^	4.72 ± 0.04 ^a^	48.10 ± 3.33 ^c^	12.21 ± 0.83 ^c^	9.15 ± 0.46 ^b^	6.43 ± 0.25 ^c^
Dried petal-macerated wine	4.18 ± 1.90 ^c^	15.33 ± 0.10 ^a^	4.67 ± 0.02 ^a^	55.36 ± 1.40 ^b^	13.51 ± 0.37 ^b^	9.24 ± 0.67 ^a^	6.76 ± 0.33 ^b^

Note: Different labeled letters in the same column of data indicate significant differences (*p* < 0.05).

## Data Availability

The original contributions presented in this study are included in the article. Further inquiries can be directed to the corresponding authors.

## Appendix A

**Table A1:** Scoring criteria and recording sheet for sensory evaluation of Dianhong rose wine.

Primary Indicator (Total Score)	Secondary Indicator	Specific Characteristic Description	Score Range
Appearance (20 points)	Color (10 points)	Deep rose red	8~10
		Medium rose red	5~7
		Pale rose red	0~4
	Clarity & Transparency (10 points)	Clear, transparent and lustrous	8~10
		Clear and transparent with no obvious suspended solids	5~7
		Slightly turbid, dull, with suspended solids and sediment	0~4
Aroma (30 points)	Rose Floral Aroma (20 points)	Intense rose floral aroma	16~20
		Faint rose floral aroma	11~15
		Insufficient or overpowering rose floral aroma	0~10
	Harmony of Floral and Wine Aroma (10 points)	Harmonious and pleasant aroma	8~10
		Relatively harmonious aroma	6~7
		Unbalanced aroma	0~5
Taste (40 points)	Palate Fullness (20 points)	Full-bodied, balanced, refreshing with lasting finish	17~20
		Moderately full-bodied, relatively balanced taste with moderate finish	14~16
		Thin-bodied, bland taste and short finish	0~13
	Honey Sweet Flavor (10 points)	Rich honey sweet note	8~10
		Faint honey sweet note	6~7
		Insufficient or overpowering honey sweet note	0~5
	Rose Flavor (10 points)	Intense rose flavor	8~10
		Faint rose flavor	6~7
		Insufficient or overpowering rose flavor	0~5
Style (10 points)	Typical Rose Wine Style (10 points)	Distinctive classic rose wine style	8~10
		Average rose wine style	6~7
		No distinctive characteristic style	0~5
